# Study of the Mechanism and Increasing Crystallinity in the Self-Templated Growth of Ultrathin PbS Nanosheets

**DOI:** 10.1021/acs.chemmater.3c00300

**Published:** 2023-03-25

**Authors:** Maaike M. van der Sluijs, Bastiaan B. V. Salzmann, Daniel Arenas Esteban, Chen Li, Daen Jannis, Laura C. Brafine, Tim D. Laning, Joost W. C. Reinders, Natalie S. A. Hijmans, Jesper R. Moes, Johan Verbeeck, Sara Bals, Daniel Vanmaekelbergh

**Affiliations:** † Condensed Matter & Interfaces, Debye Institute for Nanomaterials Science, Utrecht University, 3584 CC Utrecht, The Netherlands; ‡ Electron Microscopy for Materials Science (EMAT), NANOlab Center for Excellence, University of Antwerp, 2020 Antwerp, Belgium

## Abstract

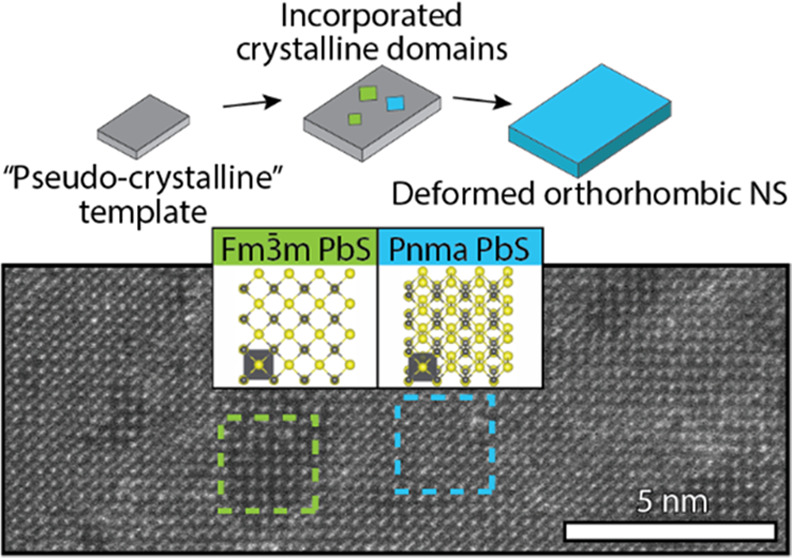

Colloidal 2D semiconductor nanocrystals, the analogue of solid-state quantum wells, have attracted strong interest in material science and physics. Molar quantities of suspended quantum objects with spectrally pure absorption and emission can be synthesized. For the visible region, CdSe nanoplatelets with atomically precise thickness and tailorable emission have been (almost) perfected. For the near-infrared region, PbS nanosheets (NSs) hold strong promise, but the photoluminescence quantum yield is low and many questions on the crystallinity, atomic structure, intriguing rectangular shape, and formation mechanism remain to be answered. Here, we report on a detailed investigation of the PbS NSs prepared with a lead thiocyanate single source precursor. Atomically resolved HAADF-STEM imaging reveals the presence of defects and small cubic domains in the deformed orthorhombic PbS crystal lattice. Moreover, variations in thickness are observed in the NSs, but only in steps of 2 PbS monolayers. To study the reaction mechanism, a synthesis at a lower temperature allowed for the study of reaction intermediates. Specifically, we studied the evolution of pseudo-crystalline templates toward mature, crystalline PbS NSs. We propose a self-induced templating mechanism based on an oleylamine-lead-thiocyanate (OLAM-Pb-SCN) complex with two Pb-SCN units as a building block; the interactions between the long-chain ligands regulate the crystal structure and possibly the lateral dimensions.

## Introduction

Two-dimensional (2D) semiconducting nanocrystals (NCs) have opto-electronic properties differing from their zero- and one-dimensional counterparts. The two long lateral dimensions result in a dispersion relation between the kinetic electron energy and momentum while quantum confinement dominates in the thin vertical direction. Hence, these systems share characteristics with epitaxial quantum wells. Moreover, in some systems, such as CdX (X = Se, S or Te) nanoplatelets, the thickness is atomically defined and these show well-defined spectral features without inhomogeneous broadening.^1,2^ Colloidal synthesis can thus result in molar quantities of solution-processable quantum objects with spectrally pure and tunable optical transitions, which is of large interest in material science and physics.^3−5^

Synthesis procedures have been developed for a broad range of 2D NCs, varying from CdSe^1,6^ and ZnSe^7^ to In_2_S_3_^8^ and PbSe,^9−11^ mainly with optical transitions in the visible spectral region. Expansion of this field toward 2D systems with efficient near-infrared emission would be beneficial for technological applications such as tissue imaging and telecommunication devices.^12,13^ Recently, progress has been made in the preparation of 2D lead sulfide nanosheets (PbS NSs).^14−20^ Intriguingly the PbS nanocrystals, naturally occurring with an isotropic cubic structure, emerge as rectangular sheets with a thickness of a few PbS units and lateral sizes in the 100 nm range. In contrast to CdSe nanoplatelets, control over the overall crystallinity, atomic structure, and electronic passivation of the top and bottom surface has not yet been achieved.^21−23^

In contrast to truncated PbS cubes, which crystallize as cubic rock salt (*a = b = c*),^24−26^ these PbS sheets show a deformed orthorhombic crystal structure (*a ≠ b ≠ c*),^17,19^ a quasi-tetragonal unit cell with bonding angles slightly different from 90°.^27^ Here, this specific crystal structure will be indicated as “deformed orthorhombic”. The common cubic crystal structure of lead chalcogenides can show a similar transition to conventional orthorhombic at elevated pressures.^28,29^ The specific origin of the deviation from the cubic crystal structure to deformed orthorhombic is unknown, but it has been proposed that the strain exerted on the crystal structure by ligands at the surface induces a phase transformation.^17,19,30^ Recent density functional theory (DFT) calculations of the bulk band structure for these crystal structures show an indirect bandgap for the conventional orthorhombic crystal structure at 0.5 eV, whereas direct bandgaps are observed for cubic and deformed orthorhombic PbS (0.74 eV).^27^ Thus, a small deformation in the crystal structure has a strong influence on the optical properties, such as the absorption and emission spectrum. Therefore, a thorough understanding of the PbS NSs crystal structure is required to understand the optical properties (or lack thereof).

Over the past few years, several synthesis methods have been reported for the preparation of PbS NSs. Early works have shown that oriented attachment and templated formation yield PbS NSs in which the 2D nanostructures are formed by the connection of small NCs.^14,31^ More recent synthesis routes have utilized single-source precursors, including lead xanthate and lead thiocyanate (Pb(SCN)_2_).^17,19^ These precursors are advantageous for upscaling,^32^ as a metal salt decomposes in an organic solvent at elevated temperatures (>150 °C), providing both the lead and sulfur atoms for the synthesis while ligands stabilize the generated NCs in situ.

In this work, we apply atomically resolved high-angle annular dark-field scanning transmission electron microscopy (HAADF-STEM) and characterize the crystal structure and defects of the sheets in detail. The intriguingly rectangular sheets have low polydispersity both in shape and lateral dimensions, despite their extended size in the 100 nm range. High-resolution imaging reveals the presence of point defects as well as cubic and conventional orthorhombic domains within the principal deformed orthorhombic crystal lattice. Moreover, we show that the sheets grow with a thickness that varies by 2 PbS units. By lowering the temperature of the synthesis, the slower reaction enables us to study the formation and evolution in the crystallinity of the intermediary reaction products by ex situ characterization. Initially, we observe rectangular pseudo-crystalline PbS templates, with a close to amorphous framework that gradually evolves into the deformed orthorhombic structure. We propose a mechanism for the formation of the PbS NSs in which an oleylamine-lead-thiocyanate (OLAM-Pb-SCN) complex, with edges that encompass 2 PbS units, is a key building block for NS formation. The surface stress exerted by the organized oleylamine capping layer is taken as responsible for the remarkable crystal structure (deformed orthorhombic) of the PbS sheets. Further analysis of the sheets grown at longer reaction times was performed with 4D STEM, a technique that scans the sample with a STEM probe in a rasterized manner and records the diffraction pattern (2D in reciprocal space) at each position on the sample (2D in real space). The dataset results in a map showing the major changes in the diffraction patterns throughout the image. Here, this analysis clearly shows a reduction in crystalline misorientations and thus an evolution toward greater crystalline homogeneity of the deformed orthorhombic PbS sheets.

## Experimental Section

### Chemicals

Acetonitrile (ACN, anhydrous, 99.8%), 1-butanol (BuOH, anhydrous, 99.8%), lead(II) thiocyanate (Pb(SCN)_2_, 99.5%), methanol (MeOH, anhydrous, 99.8%), nonanoic acid (≥97%), 1-octadecene (ODE, technical grade 90%), octylamine (99%), oleic acid (OA, technical grade 90%), and oleylamine (OLAM, technical grade 70%) were bought from Sigma-Aldrich. Toluene (anhydrous, 99.8%) was purchased from Alfa Aesar, while ethanol (EtOH, anhydrous, ≥99.8%) was purchased from VWR international.

### Synthesis of PbS nanosheets

The PbS nanosheets were prepared following a reported synthesis procedure by Akkerman *et al.*(19) In a typical synthesis, 32.3 mg (0.1 mmol) of Pb(SCN)_2_ was added to a 25 mL three-neck flask with 223.8 mg (0.250 mL) of OA, 101.6 mg (0.125 mL) of OLAM, and 7.9 g (10 mL) of ODE. The flask was capped with a Vigreux, but the reaction was allowed to proceed in air (oxygen is required for the decomposition of Pb(SCN)_2_). To dissolve Pb(SCN)_2_, the reaction flask was heated to 110 °C for 30 min, after which the temperature was quickly raised to 165 °C (at a rate of ∼15 °C per minute). Between 155 and 165 °C, the reaction mixture turns from transparent to light and then dark brown, as the temperature reaches 165 °C, and the reaction mixture in the flask was quickly cooled with a water bath (∼2 °C per second). The reaction product was washed by centrifugation at 2750 revolutions per minute (RPM, 840 g), redispersed in 5 mL of toluene, and stored in a nitrogen-filled glovebox. A possible additional washing step was performed with acetonitrile (5 mL) or MeOH/BuOH (3 mL, 1.5 mL) as an antisolvent, again centrifuging at 2750 RPM and redispersing in 5 mL of toluene. To slow the PbS NS formation down and study the reaction mechanism, the synthesis was also performed at 145 °C for up to 10 min after the color change to light brown. Via aliquots, the intermediary products were studied ex situ with HAADF-STEM and ultraviolet–visible spectroscopy (UV/Vis).

### Characterization

Transmission electron microscopy (TEM) samples were prepared by dropcasting a dilute dispersion of NCs on carbon-coated copper TEM grids. To image the atomic structure of the sheets along the planar directions and study their thickness, an antisolvent (MeOH/BuOH in a 1:2 ratio) was added to the dispersion before further sample preparation, inducing face-to-face stacking of the NSs. To diminish hydrocarbon contamination during imaging, the prepared TEM grid was treated with EtOH and activated carbon for 5 min following a recently published procedure.^33^ Bright-field TEM (BF-TEM) images were acquired with a Thermo Fisher T20, a Thermo Fisher Talos L120C, or a Fei Talos F200X operating at respectively 200, 120, and 200 keV. Low-resolution HAADF-STEM images were acquired with a Fei Talos F200X operating at 200 keV. Atomically resolved high-resolution HAADF-STEM imaging as well as 4D STEM data collection was performed on an aberration-corrected Thermo Fisher Titan G2 60-300 microscope, operating at 200 and 300 keV. The 4D STEM measurements were performed at 300 keV in microprobe mode with a convergence semi-angle of 1 mrad, resulting in a diffraction limited probe size of 0.9 nm. With this probe, the sample is scanned in a rasterized manner and a diffraction pattern is acquired at each position. The peak finding routine and virtual detector algorithms from the open-source software Pixstem are used for the analysis of these 4D STEM datasets.^34^ The datasets consist of the prove position (2D position in real space) where the pixel size is determined by the probe size, and the diffraction pattern of each pixel has been acquired (2D in reciprocal space). The final result is a map showing the major changes in the diffraction patterns, i.e., the folds in the sheets.

UV/Vis absorption spectra were measured on a PerkinElmer 950 UV/Vis/NIR spectrophotometer with quartz cuvettes. Fourier transform infrared (FTIR) spectra were measured with a Bruker Vertex 70 and an air-tight liquid cell purchased from International Crystal Laboratories, with two KBr windows and a path length of 0.5 mm. Spectra were recorded from 400 to 7000 cm^–1^ with a KBr beam splitter, a DTLaTGS D301 detector, and a mid-infrared source. FTIR spectra of PbS NS films were measured with the same apparatus and a quartz slide in an air-tight sample holder. Spectra were recorded from 3300 to 15,500 cm^–1^ with a quartz beam splitter, an Si-Diode detector, and a near-infrared source.

Proton nuclear magnetic resonance (^1^H-NMR) measurements were performed using an Agilent MRF400 equipped with a OneNMR probe and Optima Tune system. Spectra were recorded according to the following parameters: 400 MHz, CDCl_3_ 25 °C. In short, 0.6 or 0.9 mL of the synthesized PbS NSs was dried and redispersed in 0.6 mL of CDCl_3_, to which 10 μL (0.05 M) of a ferrocene stock solution was added as an internal standard. The samples were then measured with a longer relaxation delay (25 s) to allow complete relaxation.^35,36^

Atomic force microscopy samples (AFM) were prepared by dropcasting the dispersion on a cleaved mica substrate. The measurements were performed with a JPK Nanowizard 2, operating in intermittent-contact mode in an ambient atmosphere using a Bruker OTESPA-R3 tip.

## Results

### Atomically Resolved HAADF-STEM Imaging

**Figure 1:**
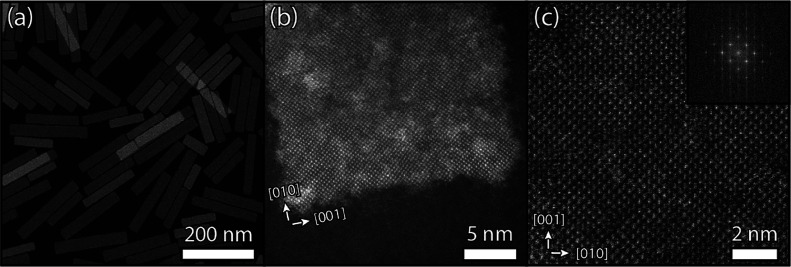
Electron microscopy images of PbS NSs prepared at 165 °C. Low-resolution HAADF-STEM images show the rectangular NSs with sharp 90° corners (a). High-resolution HAADF-STEM images show the serrated edges of the NSs (b) and a deformed orthorhombic PbS structure with dumbbell atom columns in the [100] direction (c). The inset shows the corresponding Fourier transform.

PbS nanosheets (NSs) were synthesized by the decomposition of lead thiocyanate (Pb(SCN)_2_) with oleylamine (OLAM) and oleic acid (OA), in octadecene (ODE). A procedure previously published by Akkerman *et al.*,^19^ which results in PbS NSs with well-defined geometrical features (see Figure S1a and Figure 1a), observed both in bright-field transmission electron microscopy (BF-TEM) and high-angle annular dark-field scanning transmission electron microscopy (HAADF-STEM). The sheets have a rectangular shape, straight edges, and sharp 90° corners with lateral dimensions of 142 ± 34 by 25 ± 3 nm (Figure S1b). Moreover, differences in contrast at lower magnification are observed within and between the NSs (Figure 1a,b and Figure S1c) most likely as a result of the remaining diffraction contrast or thickness differences (i.e., the number of Pb atoms in projection). With atomic resolution, the crystal structure can be studied directly (Figure 1c, Figure S2), also showing contrast differences within a single sheet.

**Figure 2:**
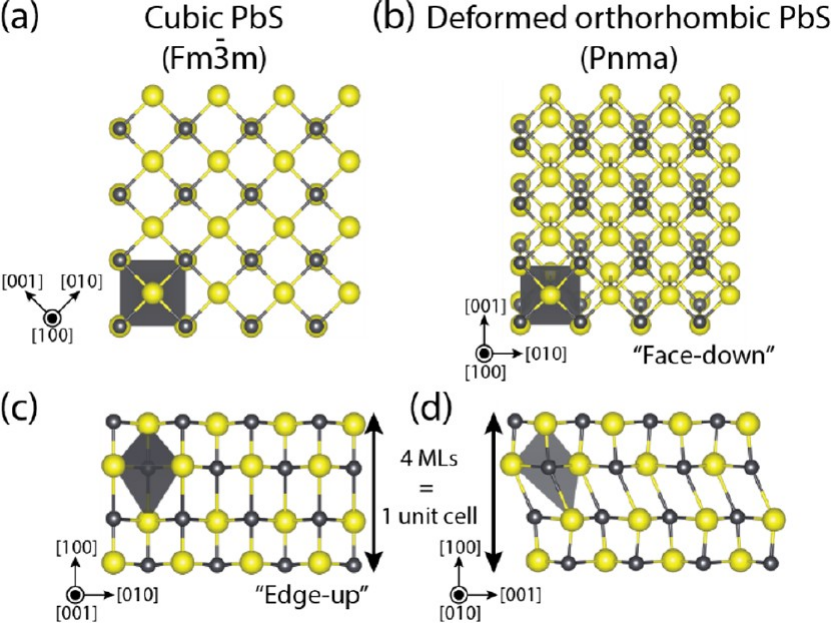
Schematic depictions of the cubic (*a* = *b* = *c*) and deformed orthorhombic PbS crystal lattices (*a* ≠ *b* ≠ *c*). The lead and sulfur atoms are represented respectively by the gray and yellow spheres. In panels (a) and (b), the face-down direction ([100] in zone axis) of both crystal structures is shown. In panels (c) and (d), the edge-up orientation on the long ([001] in panel (c)) and short edge ([010] in panel (d)) of the deformed orthorhombic NSs is shown for 4 MLs, one deformed orthorhombic unit cell. The gray octahedra clearly show that deformation occurs only in the [010] direction (d). See Figure S3 for a comparison with the cubic unit cell that consists of only 2 MLs as well as a table comparing the unit cells of the various crystal structures.

**Figure 3:**
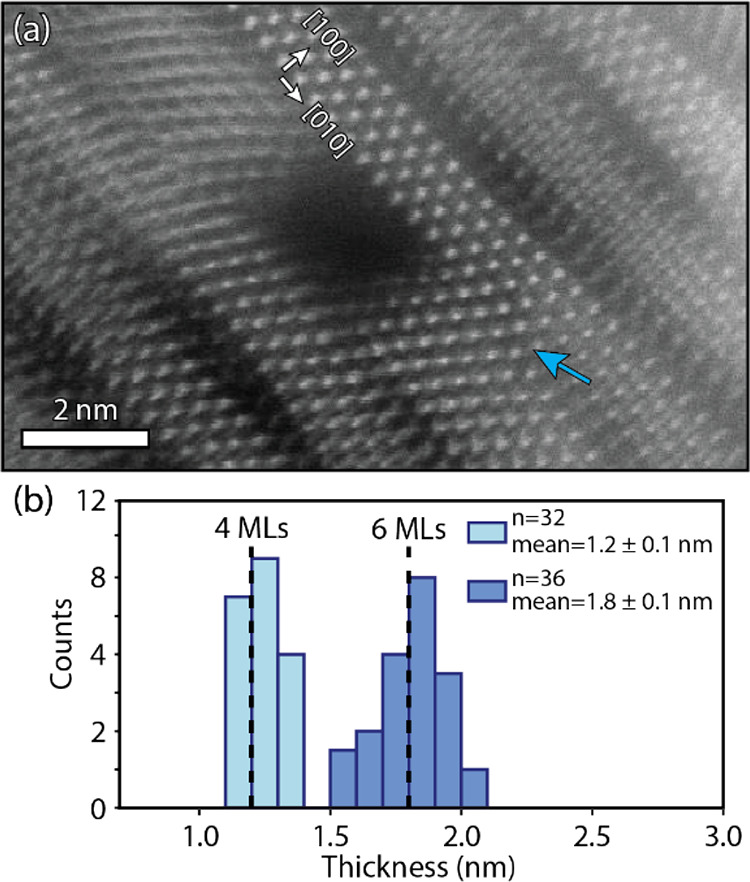
In the edge-up orientation ([001] in zone axis), the thickness of the PbS NSs can be studied with atomic precision, taking into account that some of the sheets are tilted. (a) A high-resolution HAADF-STEM image shows there are both sheets with a thickness of 4 and 6 MLs of PbS. No sheets with an intermediate thickness (3, 5, or 7 MLs) were observed. Some of the NSs merge together (blue arrow), indicating a lack of surface passivation (further discussed in Supporting Information Section 1). (b) The corresponding histogram indicates the 1:1 ratio of NSs with an average thickness of 1.2 (4 MLs) and 1.8 nm (6 MLs).

For bulk PbS and PbS nanomaterials, the isotropic cubic PbS crystal structure is common (Figure 2a).^27^ However, both bulk and nanomaterials have been shown to deform from cubic to anisotropic *conventional* orthorhombic PbS under external high pressure conditions.^28,29,37,38^ Previously, the crystal structure of the PbS NSs was assigned by fitting the powder X-ray diffraction (XRD) patterns. It was determined to be a “layered (SnS like) orthorhombic phase” with a quasi-tetragonal unit cell (*a* = 11.90 Å, *b* = 4.22 Å, *c* = 4.20 Å),^19^ which shows dumbbell-shaped atom columns in the [100] direction (Figure 2b). This is a less pronounced deformation than the conventional orthorhombic PbS structure (*a* = 11.28 Å, *b* = 4.02 Å, *c* = 4.29 Å),^27^ but with bond angles deviating from 90°. Here, we refer to the crystal structure of the NSs as “deformed orthorhombic”, as even the slight shift between the monolayers (MLs) of the unit cell induces the characteristic atomic configuration. In the face-down orientation of the NSs (see Figure 1c and 2b), the [100] direction is in zone axis, clearly displaying the dumbbell atom columns. Meanwhile, the long edge of the NSs is along the [010] axis of the deformed orthorhombic structure, whereas the [001] axis forms the short edge (Figure 2c,d and 3a).

To image the atomic structure of the sheets along the planar direction and study their thickness, an antisolvent (MeOH/BuOH in a 1:2 ratio) was added to the dispersion before further sample preparation, inducing face-to-face stacking of the NSs.^39^ With the HAADF-STEM viewing direction parallel to the sheet surface, the [001] direction is in the zone axis (Figure 2c) and the atomic columns in the edge-up orientation can be clearly observed (Figure 3a). Previously, the PbS NSs were reported to be 1.2 ± 0.3 nm thick, corresponding to 1 deformed orthorhombic unit cell of 4 MLs.^19^ Here, we observe NSs with a thickness of 4 MLs (1.2 ± 0.1 nm) but also a second equally significant population of 6 ML NSs (Figure 3b). These have a thickness of 1.8 ± 0.1 nm, similar to the conventional orthorhombic NSs previously reported by Khan *et al*.^17^ Direct observation of these two populations suggests that the contrast differences between the NSs along the face-down orientation (Figure 1a), can be attributed to the 2 MLs (0.6 nm) difference in thickness. Addition of the antisolvent decreases the NS-to-NS distance (0.8 ± 0.3 nm instead of 3.6 ± 0.3 nm^19^ or 4.4 nm^40^). At some points, the NSs even merge or grow together, indicating a lack of surface passivation (see the blue arrow in Figure 3a and Supporting Information Section 1 for an NMR and FTIR analysis of the surface passivation of the sheets).

The stepwise increase in thickness of the sheets is reminiscent of the atomic control in the thickness of zinc blende CdSe nanoplatelets. There, the top and bottom facets are terminated by Cd ligands, resulting in effective passivation and non-stoichiometry in the platelets.^41,42^ The opto-electronic properties are determined by the thickness of the NCs as the lateral dimensions are significantly larger than the exciton Bohr radius of 5.6 nm.^1,43^ This resulted in a stepwise atomic control of both the absorption and emission transitions.^6,42^ As the PbS NSs show a stepwise increase with half a unit cell (2 MLs), termination of the top and bottom facets is conventional, without an additional layer of lead. However, with an exciton Bohr radius of 23.5 nm for PbS,^44^ we expect a strong 2D confinement and qualitatively a similar relation between the thickness and the optical properties of PbS NSs (albeit with steps of 2 MLs) as observed for CdSe nanoplatelets.

The absorption spectrum of a PbS NSs dispersion with 4 and 6 MLs sheets in a 1:1 ratio lacks clear features due to scattering (Figure S7). Only the previously reported onset (usually ∼1.6 eV) is observed.^19^ By second-derivative analysis,^45^ the spectrum resolves to a single transition at 1.77 eV. Very similar to what the previously published absorption spectrum resolves to which is a single transition at 1.81 eV.^19^ Thus, we attribute the 1.77 eV transition to the 4 MLs sheets. As the reaction time increases (see below), the transition shifts to 1.64 eV (see Figure S7), which we attribute to the contribution of the NSs with 6 MLs to the absorption spectrum.

**Figure 4:**
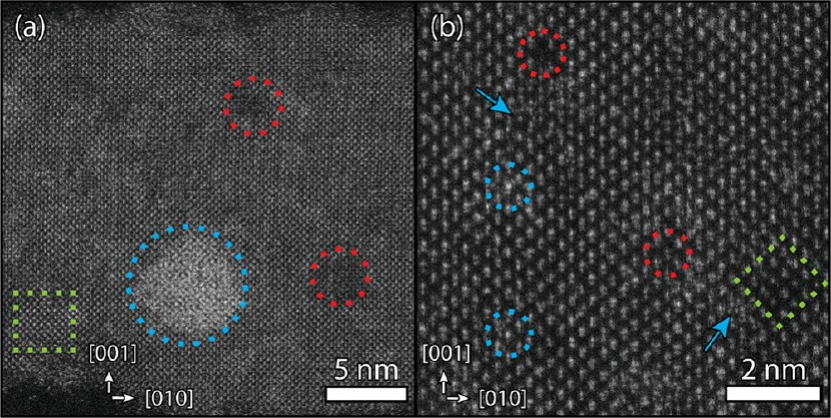
Atomically resolved HAADF-STEM images of PbS NSs, with the various irregularities in the sheets indicated by the red, blue, and green dashed outlines. (a) PbS sheet with an additional lead-rich region (blue dashed circle) and areas with a lower contrast, potentially due to a lead deficiency (red dashed circles). (b) At higher magnification, point defects (red and blue circles) and some irregularities within the structure (blue arrows) are observed. In both panels (a) and (b), a coherently incorporated small domain with a lower contrast is observed (green dashed square).

Having confirmed the presence of two thickness populations in the ensemble of PbS NSs, we studied the [100] direction of the NSs with atomic resolution. Within the deformed orthorhombic NSs, the high-contrast area (blue circle) in Figure 4a can be assigned to a lead-rich cluster (∼5 nm) attached to the top or bottom facet of the NS. Occasionally, these clusters were observed free standing in the sample as well (Figure S8). The low contrast regions (red circle) could be attributed to a collection of lead deficiencies in the crystal structure, possibly due to incomplete growth of the NS or a defect in the crystal structure, which could either be a different crystal structure or an amorphous area. Within the atom columns, similar low and high contrast is observed as well, as indicated in Figure 4b. The blue arrows indicate the presence of some point-like irregularities in the structure.

**Figure 5:**
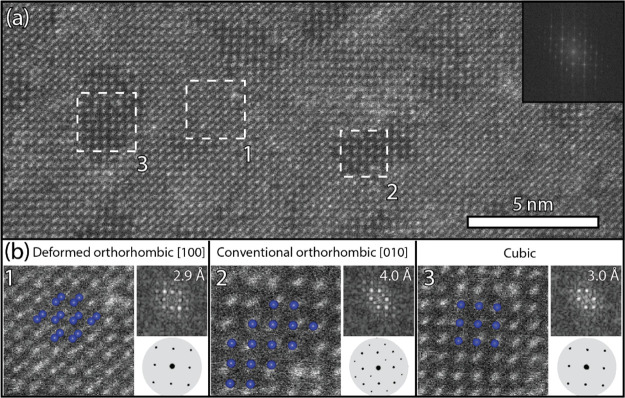
Three areas within the deformed orthorhombic PbS NS are indicated by the white dashed boxes (a), which are characterized in panel (b). The corresponding Fourier transform of domain 1 has a lattice spacing of 2.9 Å and is indexed according to the deformed orthorhombic crystal structure. Domains 2 and 3 are coherently incorporated in the principal structure, but their low-diffraction contrast and corresponding Fourier transform are different. Domain 2 is indexed as a conventional orthorhombic crystal structure with a lattice spacing of 4.0 Å along the [010] zone axis. Domain 3 is indexed as a cubic crystal structure with a spacing of 3.0 Å along the [100] axis.

In addition to the defects, the NSs also show several domains that are coherently incorporated in the deformed orthorhombic lattice but have a different crystal structure (indicated by the dashed green squares in Figure 4). The characterization of these small domains (1 to 4 unit cells) is not trivial. Figure 5 clearly shows two small domains with a different structure than the deformed orthorhombic structure. In real space, a common feature among these domains is their lower contrast with respect to the dominant crystal structure, mainly due to the absence of the characteristic dumbbell atom columns (Figure 5a). The corresponding Fourier transform patterns can be indexed according to the conventional orthorhombic (90° angles with 4.0 Å lattice spacing along the [010] zone axis) or the cubic crystal structure (with 3.0 Å lattice spacing along the [100] zone axis, Figure 5b). We hypothesize that these defects in the NSs contribute to the reported lack of photoluminescent properties.^19,27^

### Evolution of the Crystallinity of Nanosheets in Time

The deformed orthorhombic crystal structure of the PbS NSs has various crystal defects seamlessly incorporated, while sheets of both 4 and 6 MLs thick were observed in a 1:1 ratio. To understand both the origin of the deformed orthorhombic structure and the stepwise thickness increase by 2 MLs of the NSs, we studied the reaction in more detail. For colloidal 2D nanocrystals, the often suggested reaction mechanism is soft templated growth; the long organic ligands that are present are supposed to form close-packed lamellar mesophases, which could guide 2D growth.^5,31,46−50^

Relatively few studies have unambiguously shown the *in situ* presence of such soft templates during the growth of 2D nanostructures.^31,51^ An exception is PbS NSs synthesized with lead acetate, octylamine, and oleic acid, where the existence of a lamellar mesophase before nanocrystal formation was shown with low-angle XRD measurements in combination with TEM images.^31^ Another exception is the synthesis of Cu_2–*x*_S NSs, where templates observed in TEM images were tentatively described as Cu-thiolate frameworks, that maintain their 2D structural integrity at high temperatures when stabilized by halides.^51^ During the reaction, these templates gradually disappear due to Cu-catalyzed thermolysis of the carbon/sulfur bond, resulting in the formation of the NSs. For the PbS NSs studied here, it was previously proposed that a lead-oleate-thiocyanate complex forms at 110 °C, which decomposes quickly at higher temperatures yielding the PbS NSs. Even with no direct proof for the presence of templates, a similarity was drawn to the templated formation of Cu_2–*x*_S NSs.^19,51^ Here, the standard PbS NS synthesis is very fast, effectively occurring while the mixture heats up to 165 °C. However, with a lower temperature (145 °C), the reaction occurs slower. This allowed us to take aliquots and isolate intermediary reaction products to study them ex situ and gain insight into the mechanism.

**Figure 6:**
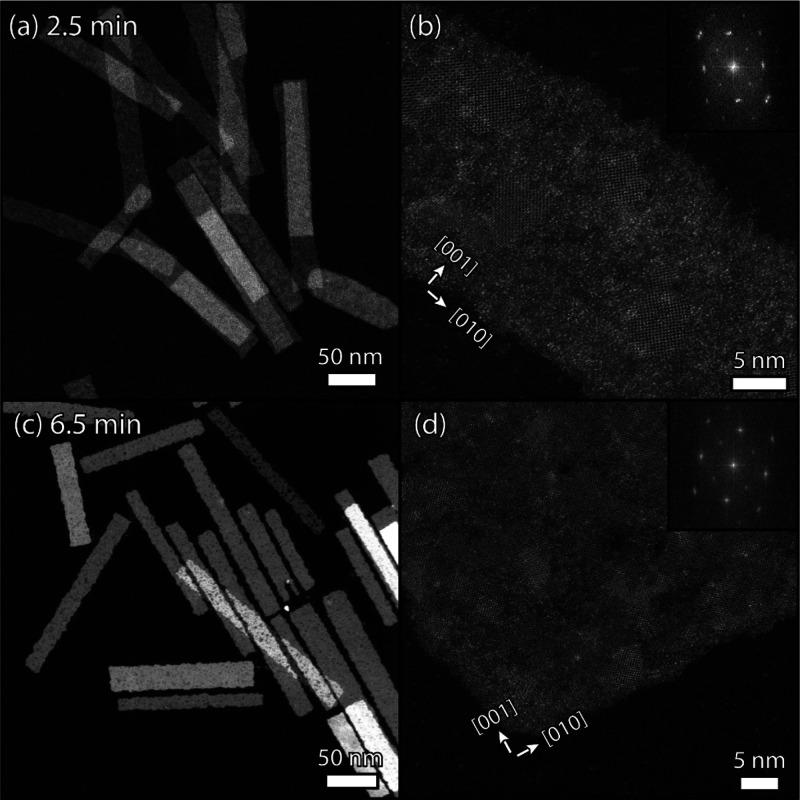
HAADF-STEM images of aliquots taken 2.5 and 6.5 min after the reaction mixture reaches 145 °C, see Figure S9 for the other aliquots. (a) Rectangular NSs with a lateral size of 160 ± 27 by 18 ± 3 nm are already present. The image with atomic resolution in panel (b) shows that the sheets are pseudo-crystalline, roughly 23% of the sheet is crystalline (on average the domain size is 25 ± 17 nm^2^, Figure S11). The Fourier transform of the sheet shows clear diffraction maxima but has some broadening in the long lateral direction. (c) After an additional 4 min, the NSs grow to an average size of 293 ± 79 by 38 ± 8 nm, while the crystallinity was estimated to be approximately 67% of the area with limited residual amorphous strips, as determined through direct visualization of the image (d).

Upon reaching 145 °C, the reaction mixture is still transparent but after 1 min and 30 s, the color changes to light brown and the first aliquot was taken. Despite the color change, no NCs were isolated (Figure S9a), but all subsequent aliquots showed rectangular NSs with 90° corners (Figure S9). Initially, these NSs have an average size of 161 by 18 nm (see Figure 6a, Figures S9b and S10a), slightly longer and thinner than the standard synthesis at 165 °C (142 by 25 nm). In time (6.5 min), the NSs grow to 293 by 38 nm with some serrated edges (Figure 6c, Figures S9d and S10b).

At higher magnification (Figure 6b), the earliest isolated PbS NSs are markedly different than the standard synthesis products at 165 °C. Instead of a crystalline structure, the sheets have an amorphous and disordered framework interspersed with crystalline areas. These domains exhibit a wide range of sizes but average 25 ± 17 nm^2^ (Figure S11), and whereas some have the deformed orthorhombic structure with the characteristic dumbbells, they primarily have one of the previously discussed defect structures. Surprisingly, the Fourier transform of the sheet shows diffraction maxima, with only some broadening in the long lateral direction (inset Figure 6b). Thus, despite the amorphous framework between them, the domains have a common orientation within the sheet. The individual deformed orthorhombic areas are oriented with the [010] axis in the long lateral direction of the NS (equivalent to the [011̅] direction for cubic PbS), just like the NSs from the standard synthesis. Even when the NSs are broken, i.e., not well defined and less rectangular, the domains are oriented (see Fourier transform inset in Figure S12). When stacked, both 4 and 6 MLs are observed in a 2:1 ratio, although these sheets are more crystalline than expected from Figure 6b (see Figures S13–S15 for a further discussion). When the NSs are kept at 145 °C for an additional 4 min, their overall crystallinity increases and only thin amorphous strips remain (Figure 6d). In absorption measurements, the optical transition gradually shifts from 1.77 eV early in the reaction (2.5 min) to 1.64 eV (Figure S16a), indicating that also the fraction of 6 ML NSs increases over time.

### Proposed Mechanism for the Growth of the PbS Nanosheets

The surprisingly rectangular NSs early in the reaction, with little crystallinity but a uniform orientation of the crystalline domains with [010] in the long lateral dimension, are an indication for a (soft) templating mechanism. Intensity profiles along the short lateral direction of this “pseudo-crystalline” NS show no significant intensity difference between the amorphous and crystalline areas (see Figure S17), suggesting a uniform thickness throughout the sheet, albeit with ordered and disordered parts. The alignment of the isolated crystalline areas and the overall increase in crystallinity of the NSs as the reaction progresses means that the initial disordered amorphous areas are arranged along the main crystallographic axes, without atomic order in the unit cells. As such, a templating mechanism as previously proposed, where the template only guides the synthesis before it disappears, does not fit with our observations.^51^ While the long chain primary amine and oleate ligands are the usual suspects for templates, the single source precursor Pb(SCN)_2_ contains the thiocyanate ion (SCN^–^), an ambidentate ligand. Hence, the pseudo-crystalline template, which crystallizes and forms the sheet, can only form with the involvement of the single source precursor.^52^ Here, we propose a self-induced template that imposes a 2D constraint during the reaction but is also part of the eventually formed NSs.

As it is ambidentate, the thiocyanate ion can form coordinating bonds with a metal ion (M) via sulfur (S) or nitrogen (N) via their unshared electrons. N-bonding is favored when a borderline or hard acid is present whereas a soft acid will induce S-bonding, resulting in M-NCS or M-SCN complexes, respectively. In the case of a borderline acid (such as Cd^2+^ and Pb^2+^), both complexes can be formed and the electronic and steric factors of other ligands bound to the metal center become very influential.^53−55^ Here, OA and OLAM are present during the decomposition of Pb(SCN)_2_ and as both ligands can coordinate with lead, the electron density on the lead atom can vary. In a complex with OA (an X-type electron donating/accepting ligand),^36,56^ the electron density on the lead ion is lower, resulting in an OA-Pb-NCS complex. However, in a complex with OLAM (an L-type electron donating ligand),^57^ the lead ion will have an increased electron density, and an OLAM-Pb-SCN complex can form.^55^

Previously, the presence of excess ligands during the reaction was shown to result in polydisperse NSs or cubic nanocubes.^19^ However, with an equivalent amount of only OA, no discernible nanocrystals are formed (Figure S18a), while with only OLAM 2D structures and some cubes form (Figure S18b). This indicates the importance of an amine in this reaction. Additional experiments with a shorter acid and amine ligand (nonanoic acid and octylamine, Figure S19) showed that the variation in the width of the NSs increases significantly with octylamine. Instead of the usual 25–35 nm width (also achieved with nonanoic acid Figure S19c), the resulting NSs had an average of 130 nm with standard deviation of 60 nm (Figure S19d). Thus, a shorter amine ligand allows more freedom in the formation of the sheets, whereas shorter acidic ligands still result in NSs with a similar constraint in width.

**Figure 7:**
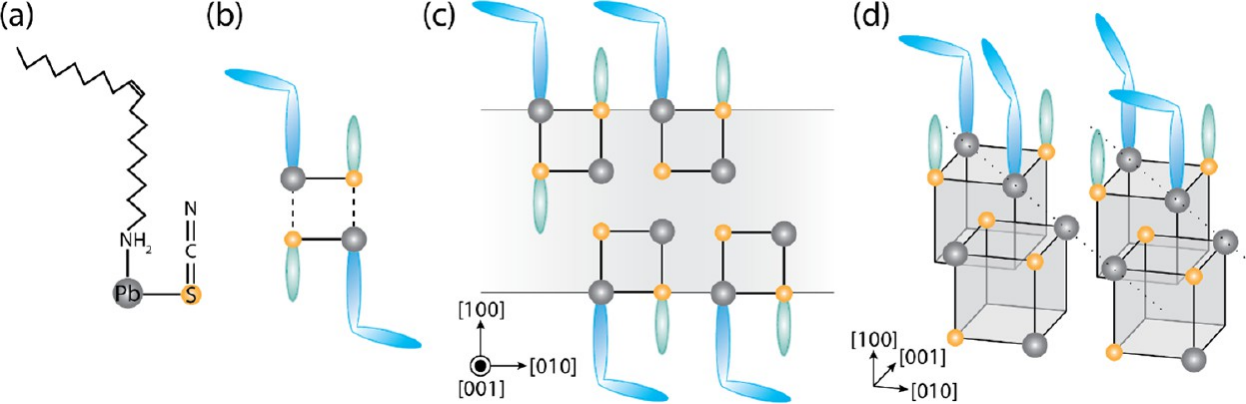
Schematic formation of PbS NS via a self-induced templating mechanism. Starting with an OLAM-Pb-SCN complex with a 90° angle between the Pb/S and C/S bonds (a). By cross-coordination, two OLAM-Pb-SCN complexes can form a square, the central building block of the self-templating mechanism (b). Two-dimensional formation of the pseudo-crystalline structure as observed in the edge-up orientation of the PbS NS (c). Growth of the NSs along the lateral directions via cubes (four cross-coordinated OLAM-Pb-SCN complexes) with diagonal alignment of the favorable ligand–ligand interaction resulting in the [011̅] orientation of the lead atoms (d).

Hence, we propose that an OLAM-Pb-SCN complex is the precursor to the central building block for a self-induced template resulting in PbS NSs. An amine group bound to the lead ion will induce S site bonding of the thiocyanate, already placing the lead and sulfur in proximity (Figure 7a). In this complex, the angle between the lead/sulfur (Pb/S) bond and the carbon/sulfur (C/S) double bond is expected to be around 90°, previously shown to be the case for both cadmium-SCN and zinc-SCN complexes.^55^ The angled complex allows ample space for coordination with another complex, resulting in a building block with 2 Pb–S units (Figure 7b), in line with the 2 ML stepwise increase in the NSs thickness (Figure 3). As no NSs consisting of 2 MLs were observed in the experiments, we consider these building blocks too unstable to form NSs with an ionic lattice. However, as they interact with other building blocks, a system with 4 Pb–S units begins to form. Depending on the bond strength of the ligands, and the C/S double bond, cross-correlation begins, eventually forming the basis for the ionic lattice of the NSs (Figure 7c). The pseudo-crystalline NSs in Figure 6b can be described by this part of the mechanism. Favorable ligand–ligand interaction occurs as the building blocks are roughly organized with an [011̅] orientation of the lead atoms, corresponding to an [010] orientation of the deformed orthorhombic structure. However, the building blocks are not yet fully crystallized, i.e., the C/S double bond has not yet broken and the PbS lattice not yet formed in the entire template. In this mixed system, containing both pseudo-crystalline and crystalline parts, the strain induced by the strong ligand–ligand interaction can still be distributed throughout the entire system. It does not yet affect the smaller predominantly cubic crystalline areas. Upon continued thermal annealing, the bonds in the building blocks break and the ionic lattice forms (Figure 7d). The favorable diagonal long-range organization of the ligands increases, resulting in an increased surface tension that can no longer be accommodated in the ultrathin PbS sheets, thus inducing the cubic to deformed orthorhombic transition of the crystalline domains.

The mechanism described above is a self-induced templating mechanism in which the basic building blocks already favorably interact with each other, helped along by their templating long-chain organic ligands. Upon further annealing, the bonds in the building block break resulting in the formation of the ionic PbS lattice.

### Improving the Crystallinity of the Nanosheets

With the crystalline structure of the NSs and its defects characterized, the thickness of the NSs studied and our understanding of the mechanism increased, we set out to improve the NSs. As previously discussed, the sheets have not yet shown any photoluminescence, not even after chloride surface treatments,^19^ presumably due to the intrinsic defects in the sheets synthesized by the standard synthesis, a fast decomposition reaction that is quenched when reaching 165 °C. By prolonging the reaction at 165 °C for 5, or even 10 min, we effectively annealed the NSs to improve their crystallinity.

**Figure 8:**
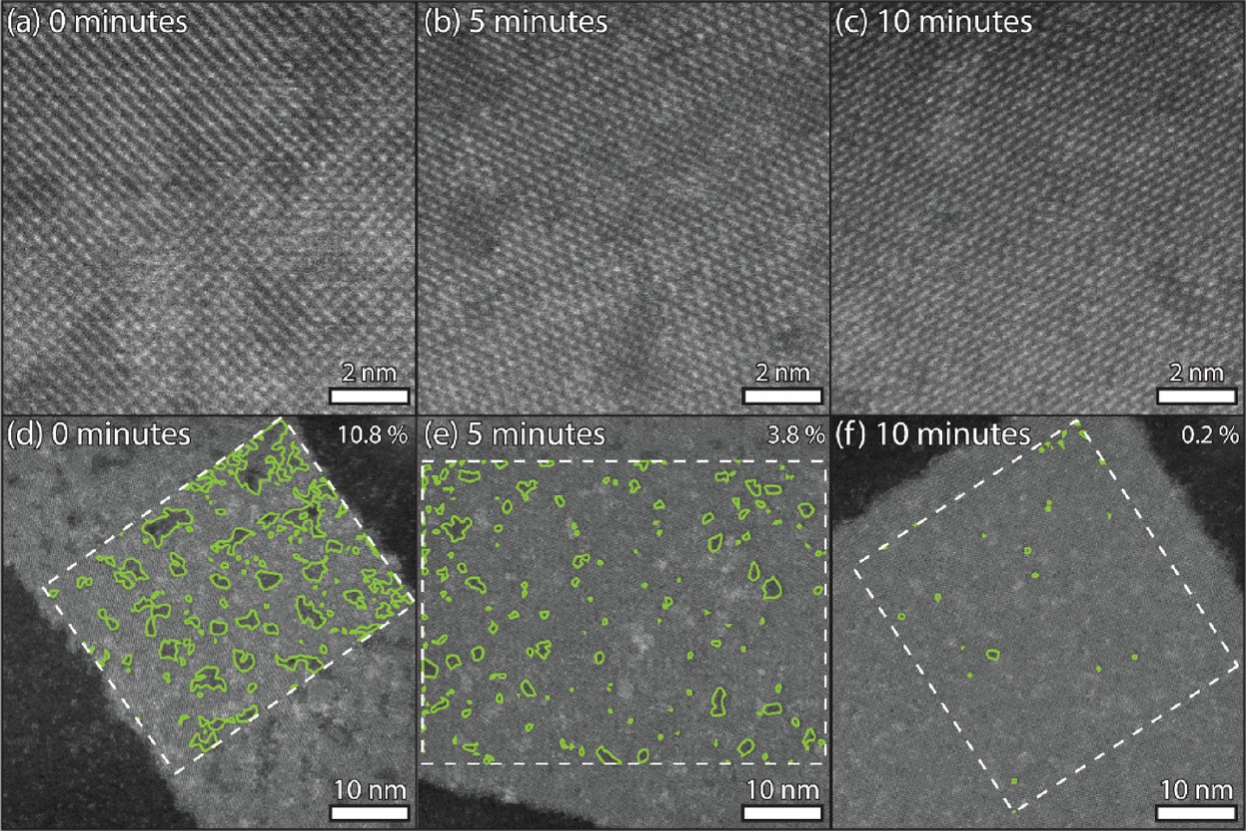
When annealing the NSs at 165 °C for an additional 5 or 10 min, the uniformity of the deformed orthorhombic crystal structure increases (a–c). With intensity threshold analysis applied to the low-contrast defect areas in the NSs (indicated by a green outline), a quantitative estimation on the improvement can be made. The defect areas decrease from 10.8% to 3.8% and then 0.2% (c–e).

Figure 8b shows an HAADF-STEM image of a PbS NS annealed for 5 min at 165 °C, still clearly with the dumbbells of the [100] direction of the deformed orthorhombic crystal structure and some low-contrast areas. Based upon their Fourier transform, these areas are characterized as different crystal structures such as cubic and conventional orthorhombic (Figure S20). Although the crystal structure becomes more uniform after an additional 5 min (Figure 8c), there are still some defects present (Figure S20b). At the same time, the previously observed 1:1 ratio of 4 and 6 MLs thick NSs (Figure 3b) shifts firmly to 6 MLs (see Figure S21). In adsorption spectra of the dispersion, the scattering increases while the transition becomes more pronounced. The second-derivative analysis shows a transition at 1.64 eV, indicating that the NSs predominantly have a thickness of 6 MLs (Figure S22a). When the absorptance of a PbS NSs film was measured (Figure S22b), the absorptance increased from ∼10% to ∼40%, indicating an increase in the overall concentration of fully crystalline NSs.

Considering that the dumbbell atom columns of the deformed orthorhombic structure give the NSs higher intensity at low magnification with respect to the defect areas, we can apply intensity threshold image analysis. This allows for the tentative estimation of the defect percentage in the sheets. Synthesized by the standard procedure, they show up to 10.8% defect areas in a 25 by 35 nm area, as indicated by the green outlines in Figure 8d. As the NSs are annealed at 165 °C for 5 or 10 min, the defect percentage decreases to 3.8 and 0.2%, respectively (Figure 8e,f).

While an indication that the areas with a lower diffraction contrast are quite common, a technique such as 4D STEM can be of tremendous help in visualizing homogeneity of the crystal orientations in the sheets. With a STEM electron probe, the sample is scanned in a rasterized manner and at each position a diffraction pattern is acquired. The result has been interpreted as an HAADF-STEM image (2D in real space) where the pixel size is determined by the probe size and where the diffraction pattern of each pixel is known (2D in reciprocal space, Figure S23). 4D STEM was utilized to map changes in the reciprocal space of the sheets, effectively displaying the enhancement in crystal orientation as synthesis time increases.

**Figure 9:**
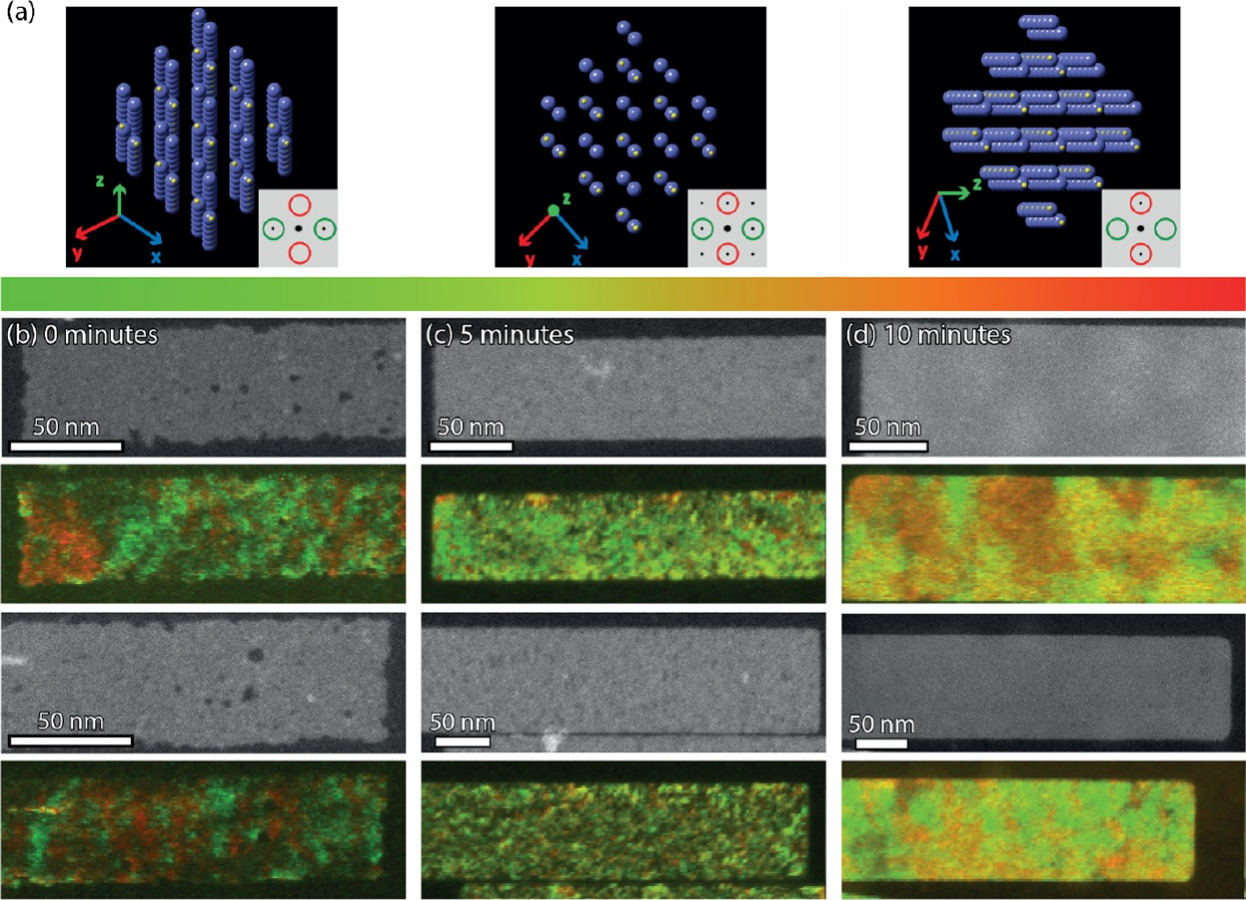
With 4D STEM the changes in orientation of crystal structure can be tracked in the PbS NSs. (a) The deformed orthorhombic crystal structure is schematically depicted as it tilts in the short (left) and long (right) lateral direction of the sheet. With the [100] direction in zone axis, the diffraction pattern shows 4 spots with a lattice spacing of 0.29 nm contributing equally (middle image inset, yellow color). As the structure tilts, the contribution of the diffraction maxima changes and each pixel in the 4D STEM image was characterized based on the contribution of the diffraction maxima, green for a tilt in the short lateral direction and red for the long lateral direction. The HAADF-STEM image, and its reconstructed 4D STEM map are shown for 0 min (b), 5 min (c), and 10 min (d) at 165 °C. In panel (b) NSs immediately quenched upon reaching 165 °C have some voids in the HAADF-STEM image, while the maps also show large regions with different parallel orientations in the NSs. A significant improvement in the tilt toward zone axis can be observed in panel (b) and even more in panel (c) where large homogeneous areas of dark yellow can be observed.

When characterizing each pixel in the HAADF-STEM image based upon the intensity of the {011} family planes in the diffraction pattern (at a lattice spacing of 0.29 nm), the changes in orientation of the crystal structure can be tracked. Figure 9 displays the color maps generated by assigning red or green to the intensities of diffraction maxima on perpendicular distances. When the structure is imaged along the zone axis (Figure 9a, middle image) the map appears dark yellow, as the red and green spots contribute equally to the overall intensity. If the structure is tilted along a certain direction, one of the intensities will decrease, resulting in a red or green color (Figure 9a, left and right). The corresponding maps in Figure 9b–d show the folded areas in the NSs. The NSs immediately quenched upon reaching 165 °C (Figure 9b) show some voids in the maps, some of which are also observed in the HAADF-STEM image. Otherwise, they correspond either to a possible dealignment with the main crystal structure or the presence of the small domains discussed above with different crystal structures. Initially, the thinner NSs have large folds where the structure is tilted in one direction, right next to areas tilted in the other direction, i.e., green right next to red areas (Figure 9b). However, as the NSs are effectively annealed for longer times, the folds become less prominent; hence, as the NSs become thicker (6 MLs), they also become more rigid (Figure 9c,d) and their crystal structure improves.

## Conclusions

In summary, atomically resolved HAADF-STEM imaging was used to characterize the deformed orthorhombic PbS NSs, both during their formation and as final products. The 2D ultrathin sheets allowed for observation of contrast differences within and between the sheets. When stacked, two populations of 4 and 6 MLs were observed in a 1:1 ratio (1.2 and 1.8 nm). In absorption spectra, strong light scattering obscures the optical transactions, but analysis with the second-derivative elucidates an optical transition at 1.77 eV for 4 MLs and 1.64 eV for 6 MLs thick NSs. This is far above the gap of PbS bulk and quantum dots with a cubic crystal structure. With atomic resolution, the subtly incorporated low-contrast crystal defects stand out; by Fourier analysis, these specific areas could be characterized as cubic or conventional orthorhombic areas.

By studying the intermediary products of the reaction at a lower temperature (145 °C), we gained insight into the reaction mechanism. Initially, a pseudo-crystalline rectangular template is formed, consisting of amorphous and crystalline areas, which are uniformly oriented with the [010] axis in the long lateral dimension. We propose that these PbS NSs form via a self-induced templating mechanism, in which the single source precursor Pb(SCN)_2_ plays a key part. We suspect that an OLAM-Pb-SCN complex initiates the 2D formation of the self-induced template. Favorable ligand–ligand interaction of the complexes induces the [010] orientation, even before the formation of an ionic lattice in these pseudo-crystalline templates. The surface strain induced by the long OLAM ligands on the ionic lattice induces the deformed orthorhombic crystal structure when the crystallinity in the sheet increases. Only some additional thermal annealing is needed to form a deformed orthorhombic PbS lattice from the pseudo-crystalline template.

A further reduction of the number of intrinsic crystal defects, edge imperfections, and lead-rich clusters will be required to eliminate the non-radiative recombination channels in the NSs. To improve the crystallinity of the sheets, continued reaction at high temperature (5 or 10 min at 165 °C) proved to be quite effective. The number of crystal defects in the sheets decreases significantly, resulting in almost perfect deformed orthorhombic PbS NSs predominantly with a 6 MLs thickness. The 4D STEM technique effectively demonstrates a reduction in both occurrence and severity of crystal misorientations in the sheets over the course of annealing and therefore an increased crystallinity over synthesis time. Obtaining PbS sheets with a respectable opto-electronic performance will be a challenge since the radiative life-times in the near-infrared are relatively large compared to the visible region. However, it will be worth the effort as the deformed orthorhombic crystal structure, the related uncommon electronic band structure, and the two-dimensionality offer a pathway to novel 2D semiconductors with a bandgap in the interesting energy region just below the visible.

## Supporting Information Available

Additional BF-TEM and HAADF-STEM images including characterizing measurements; high-resolution BF-TEM image showing the atom columns in the [100] direction; schematic depiction of cubic PbS crystal structure and table comparing the various unit cells of PbS; section discussing the NMR and FTIR study of additional purification steps; absorption spectra and corresponding second-derivative analysis of a standard PbS NSs synthesis; high-resolution HAADF-STEM images showing free-standing clusters; section showing the additional study of the NSs synthesized at 145 °C including HAADF-STEM images of each sample and characterizing measurements, average crystalline domain size within the 2.5 min sheets and HAADF-STEM images of “broken” NSs; HAADF-STEM images and analysis of sheets stacked by antisolvent addition; AFM measurements of the NSs early in the reaction; histograms of the thickness measured by AFM and on unintentionally stacked NSs in TEM; absorption spectra of aliquots from a 145 and 160 °C reaction with the corresponding second-derivative analysis; intensity profile of the pseudo-crystalline NSs; section with additional study of the formation mechanism, showing representative HAADF-STEM images of syntheses with only oleic acid and only oleylamine, HAADF-STEM images and analysis of the reaction products with nonanoic acid and octylamine; section with additional analysis of the annealed NSs including defects, thickness and optical properties; schematic representation of the diffraction patterns of various crystal structures (PDF)

## Abbreviations

2D: two-dimensional
4D STEM: scanning transmission electron microscopy subset which captures a 2D reciprocal space image associated with each scan point of a raster in a 2D region in real space
AFM: atomic force microscopy
BF-TEM: bright-field transmission electron microscopy
DFT: density functional theory
FTIR: Fourier transform infrared spectroscopy
HAADF-STEM: high-angle annular dark-field scanning transmission electron microscopy
MLs: monolayers
NCs: nanocrystals
NMR: nuclear magnetic resonance
NSs: nanosheets
OLAM-Pb-SCN complex: a complex from oleylamine and the thiocyanate ion bound to lead
RPM: revolutions per minute
UV/Vis: ultraviolet–visible spectroscopy
XRD: X-ray diffraction
