# Supplementary material for: Study of the Mechanism and Increasing Crystallinity in the Self-Templated Growth of Ultrathin PbS Nanosheets
Source: Chem Mater. 2023 Mar 25;35(7):2988–98. doi: 10.1021/acs.chemmater.3c00300 (PMC10100538; doi:10.1021/acs.chemmater.3c00300)
Supplement: Supplementary file 1 — cm3c00300_si_001.pdf [file cm3c00300_si_001.pdf]

Study of the Mechanism and Increasing Crystallinity in the Self-Templated Growth of Ultrathin PbS Nanosheets

# – Supporting Information

Maaike M. van der Sluijs,[1] Bastiaan B. V. Salzmann,[1] Daniel Arenas Esteban,[2] Chen Li,[2] Daen Jannis,[2] Laura C. Brafine,[1] Tim D. Laning,[1] Joost W. C. Reinders,[1] Natalie S. A. Hijmans,[1] Jesper R. Moes,[1] Johan Verbeeck,[2] Sara Bals[2] and Daniel Vanmaekelbergh[1]*

1 Condensed Matter & Interfaces, Debye Institute for Nanomaterials Science, Utrecht University, 3584 CC Utrecht, The Netherlands.

2 Electron Microscopy for Materials Science (EMAT), NANOlab Center for Excellence, University of Antwerp, 2020 Antwerp, Belgium.

*Corresponding author: d.vanmaekelbergh@uu.nl.

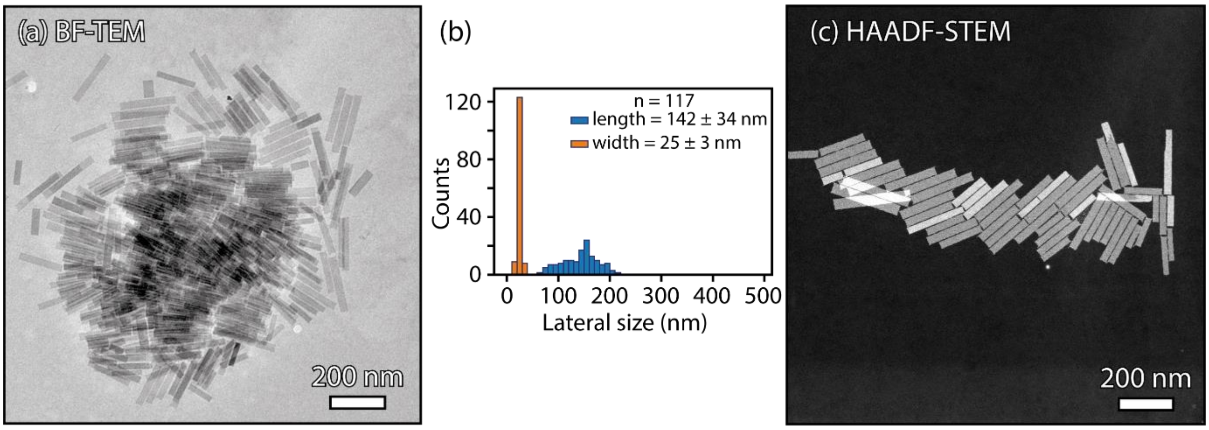

Figure S1. Overview images of a standard synthesis of PbS NSs prepared at 165 °C imaged with BF-TEM (a) and HAADF-STEM (c). The NSs in the TEM images were measured with ImageJ and the histogram shows the lateral dimensions (with average 142 ± 34 by 25 ± 3 nm). The low-resolution HAADF-STEM image of a standard synthesis of PbS NSs (c), shows contrast differences between the NSs.

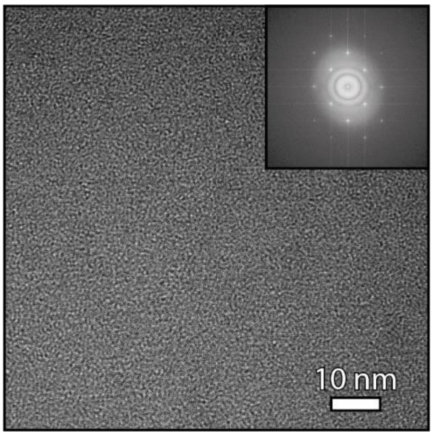

Figure S2. High-resolution BF-TEM image showing the atomic columns along the [100] direction in a face-down oriented PbS NS with the corresponding Fourier transform. While the atom columns are visible, additional contrast from the formvar/carbon background makes in-depth characterization of the crystal structure difficult. Therefore, atomically resolved HAADF-STEM is preferred over BF-TEM, see also Figure 1c.

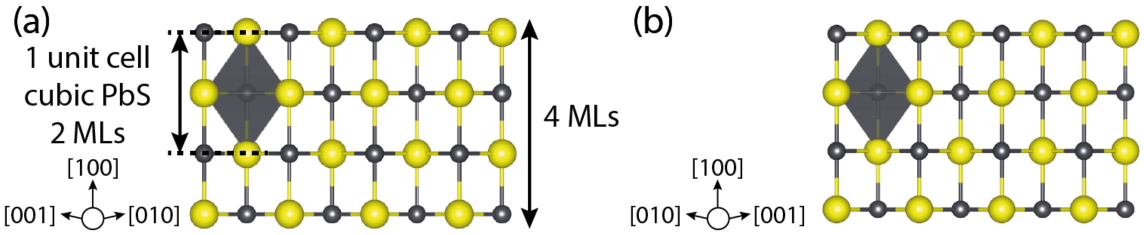

(c)

Unit cells	ICSD	a (Å)	b (Å)	c (Å)	Bandgap type
Cubic	5557	5.91	5.91	5.91	Direct
Deformed orthorhombic	14945	11.90	4.22	4.20	Direct
Conventional orthorhombic	648451	11.28	4.02	4.29	Indirect

Figure S3. Schematic depiction of cubic PbS, as it would show on the side of the NSs if they had a cubic structure (a and b). The grey and yellow symbols represent the Pb and S atoms respectively. As the cubic crystal structure is isotropic, no differences are observed between the long edge (a) and the short edge (b) of the NSs. In panel (c) the unit cell dimensions of the various crystal structures discussed here are shown. Including the bulk bandgap type as calculate by DFT using the unit cells in the table. Showing that the small increase in lattice anisotropy from deformed orthorhombic to conventional orthorhombic is enough to switch the calculated bulk band gap from direct to indirect.[1]

Section S1. NMR and FTIR study of additional purification steps

After addition of an antisolvent to induce stacking of the NSs, the NS-to-NS distance decreases (which is 0.8 ± 0.3 instead of 3.6 ± 0.3 nm[2] or 4.4 nm[3]). At some points the NSs even merge or grow together, indicating a lack of surface passivation (see the blue arrow in Figure 3a). To gain some understanding of the residual precursors in the dispersion of the sheets and to study the surface passivation, we used ^{1}H-NMR measurements. NSs purified by centrifugation and redispersion in fresh toluene (the purification steps previously reported),[2] show the presence of the organic ligands and ODE (Figure S4). The characteristic broadening of the 5.3 ppm peak for surface bound OA and/or OLAM is not observed.[4-6] Additional purification by the addition of a protic or aprotic antisolvent removes most of the residual organic ligands and ODE (see Figure S5). After these purification procedures, the thiocyanate is still observed at ~2040 cm^{-1} with FTIR (see inset supplementary Figure S6). We therefore suspect that the residual organic ligands are removed in the stacked NSs, resulting in the decrease of NS-to-NS distance (Figure 3a).

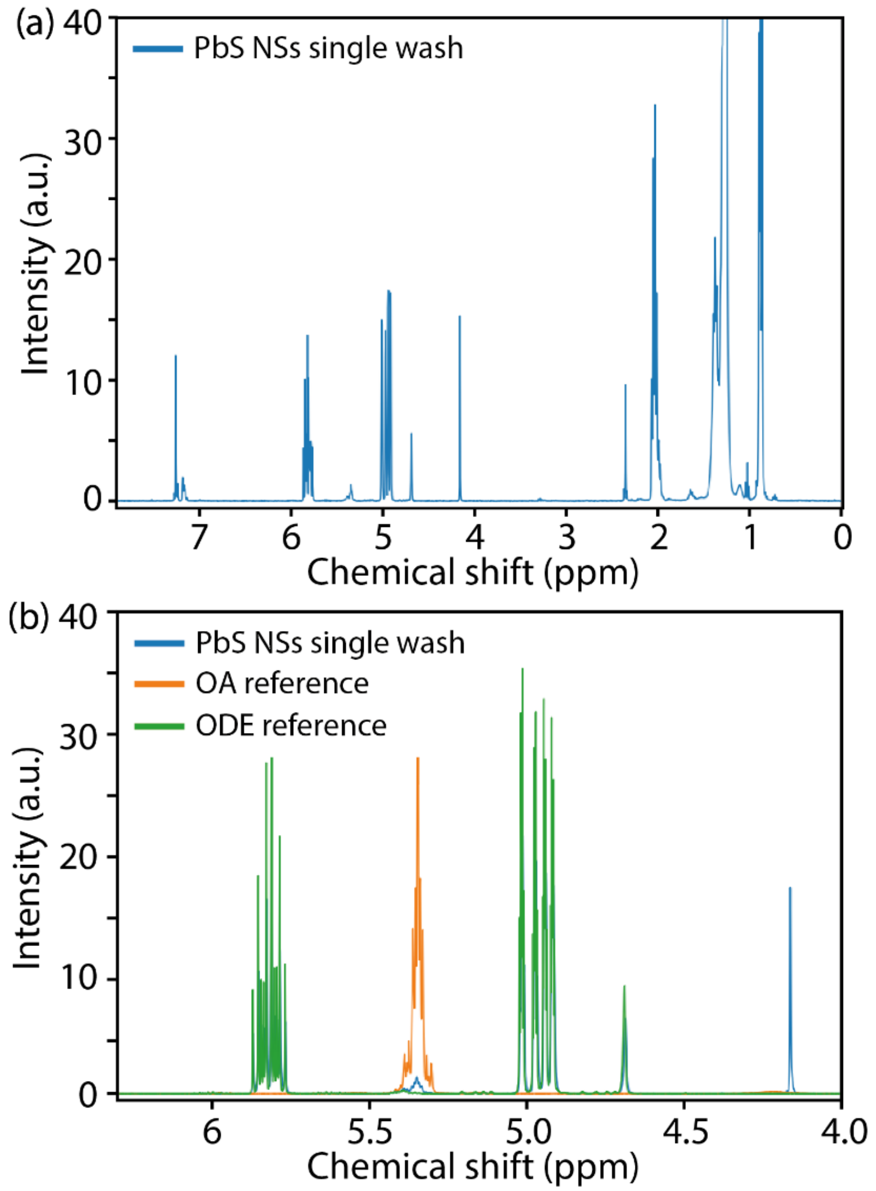

Figure S4. NMR spectra of PbS NSs and references. (a) NMR spectrum of PbS NSs, see Table S1 for a full reference of each peak. (b) Selection of the NMR spectrum in (a) with reference spectra of OA and ODE. The sharp resonances at 5.8 ppm and 4.9 ppm are attributed to ODE, the 5.3 ppm resonance is attributed to the presence of OA/OLAM, although the characteristic broadening surface bound ligands, previously observed both in 3D and 2D materials is not observed here.[4-6]

Table S1. Table listing the resonances observed in Figure S4a and attributed to the protons present in the reaction mixture. Note that thiocyanate does not contain any protons and therefore cannot be characterized with ^{1}H-NMR.

**Chemical shift (ppm)**	**Corresponds to**	**Group**
0.9	OA, ODE, OLAM	- C**H$_3$**
1.3	OA, ODE, OLAM	-C**H$_2$**
2.0	OA, ODE, OLAM	-C**H$_2$**, -N**H$_2$**
2.3	OA	O=C—C**H$_3$**
2.7	OLAM	-C**H$_2$**-NH$_2$
4.1	Ferrocene	-
4.9	ODE	=C**H$_2$**
5.3	OA, OLAM	-**H**C=C**H**-
5.8	ODE	-C**H**=CH$_2$
7.3	$CDCl_3$	-

In Table S1, a complete overview of every resonance and their characterization is given. The concentration ODE and OA/OLAM were determined by the addition of a known concentration of ferrocene (10 µL, 0.05M).[4,7] The concentrations ODE and OA/OLAM were determined using the internal standard with the following equations:

$$[\mathrm{ODE}] = \left(\frac{I_{4.9}}{I_{fer}}\right) * \left(\frac{10}{2}\right) * [ferrocene]$$

$$[\mathrm{OA/OLAM}] = \left(\frac{I_{5.3}}{I_{fer}}\right) * \left(\frac{10}{2}\right) * [ferrocene]$$

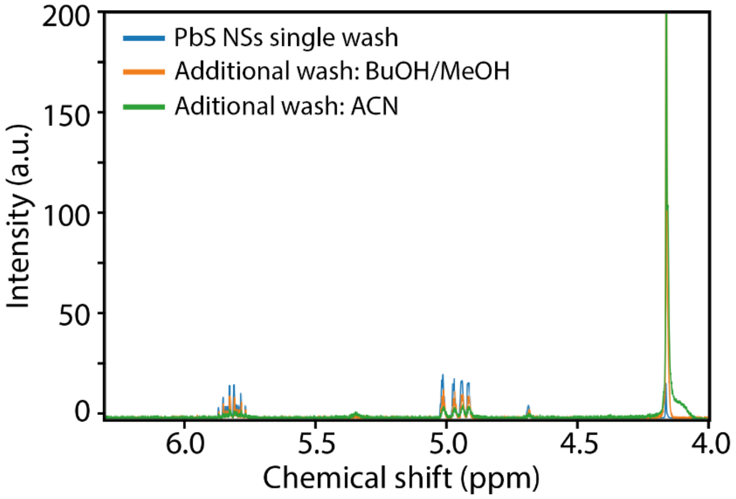

Figure S5. NMR spectra of PbS NSs centrifuged and redispersed (blue) and washed with two different antisolvents (orange, green). Ferrocene was added as an internal standard and used to calculate the concentration of ODE and OA/OLAM in the sample.

Table S2. A table listing the integrals of the NMR spectra shown in Figure S5. Values used to calculate the concentration of the corresponding molecules present in each sample. After additional washing steps with an antisolvent, the concentration has significantly decreased.

Sample	Integral 4.1 ppm (ferrocene)	Integral 4.9 ppm (ODE)	Integral 5.3 ppm (OA/OLAM)	Concentration ODE [µM]	Concentration OA/OLAM [µM]
PbS single washed	1	11.81	0.58	45*	2.2*
PbS Me/Bu	1	0.23	0.01	0.58	0.025
PbS ACN	1	0.13	0.03	0.33	0.075

*Scaled by a factor of 1.5 for comparison with other samples, as only 0.6 mL sample was measured instead of 0.9 mL.

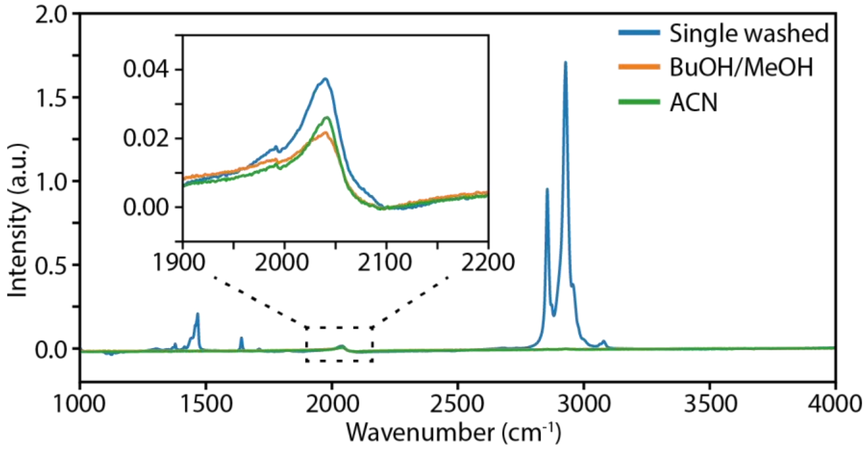

Figure S6: FTIR spectra of PbS NSs centrifuged and redispersed (blue) and washed with two different antisolvents (orange, green). Showing the presence of thiocyanate group even after further purification.

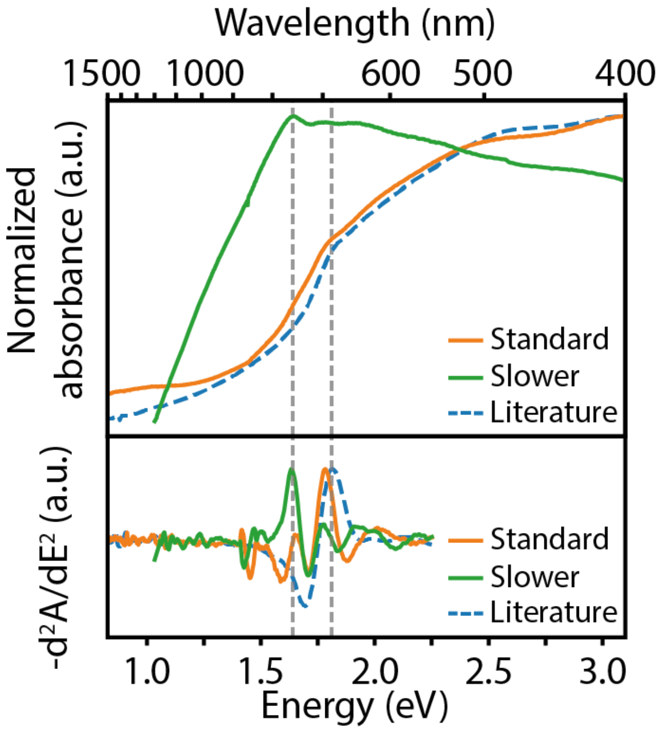

Figure S7. Absorption spectra and corresponding second-derivative analysis of a standard synthesis (standard 165 °C, orange), a synthesis with a heating rate of 9 °C per minute (slower 165 °C, green) and previous results of Akkerman et al. (suspension in Figure 4a digitized,[2] dashed blue). The standard sample contains a 1:1 ratio of both 4 and 6 MLs thick PbS NSs, the second-derivative analysis shows a single maximum at 1.77 eV. A maximum of 1.81 eV is observed for the literature absorption spectrum with only a 4 MLs population. Despite the presence of two populations, the absorption features are dominated by the optical transition of the 4 MLs NSs. In the slower synthesis the second-derivative shows a single maximum at 1.64 eV, presumably due to a majority of 6 MLs NSs. The grey dashed lines are a guide to the eye at 1.64 eV and 1.77 eV.

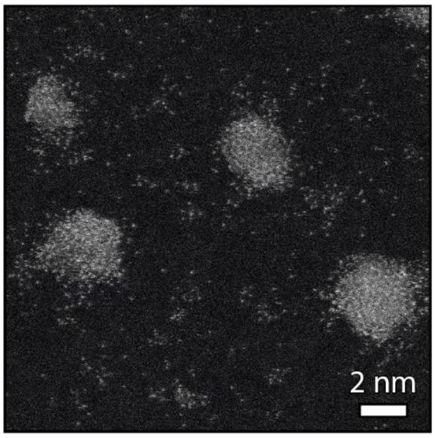

Figure S8. High-resolution HAADF-STEM image of free standing clusters roughly 2 nm in size. Their lack of a well-defined crystal structure is similar to previous literature on CdSe nanocrystals, where nanoclusters with a decreased size (below 2 nm) were shown to lack crystallinity.[8]

Section S2. PbS NSs synthesized at 145 °C

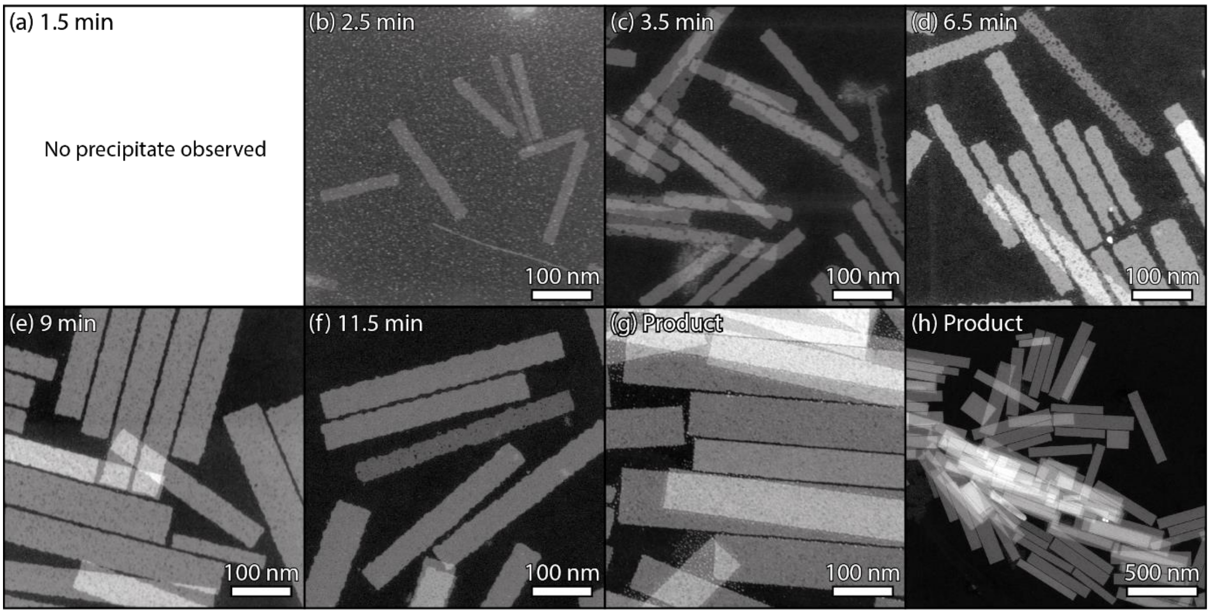

Figure S9. HAADF-STEM images of the aliquots taken at 145 °C. Time zero is defined as the moment that the reaction mixture reached 145 °C, the first aliquot was taken the moment that the characteristic color changes from transparent to light brown and did not yield any precipitate (a). All other intermediary products were isolated and studied with HAADF-STEM continually showing the presence of PbS NSs at increasing sizes (b-h).

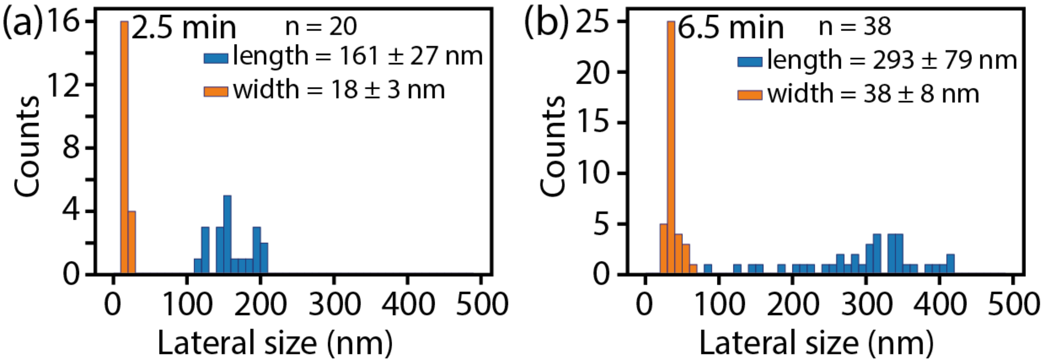

Figure S10. Lateral dimensions of PbS NSs prepared at 145 °C after 2.5 minutes (a) and 6.5 minutes (b) of reaction. Show an overall increase from 161 ± 27 by 18 ± 3 nm to 293 ± 79 by 38 ± 8 nm. With a large increase of the standard deviation, although it should be noted that the number of measured NSs is quite low, 20 and 38.

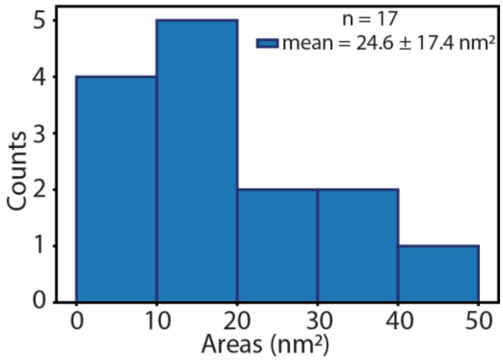

Figure S11. The crystalline domains in the pseudo-crystalline sheets synthesized at 145 °C for 2.5 minutes were measured with ImageJ. The histogram clearly indicates a large range in size, resulting in an average of 24.6 nm^2 but with a standard deviation of 17.4 nm^2.

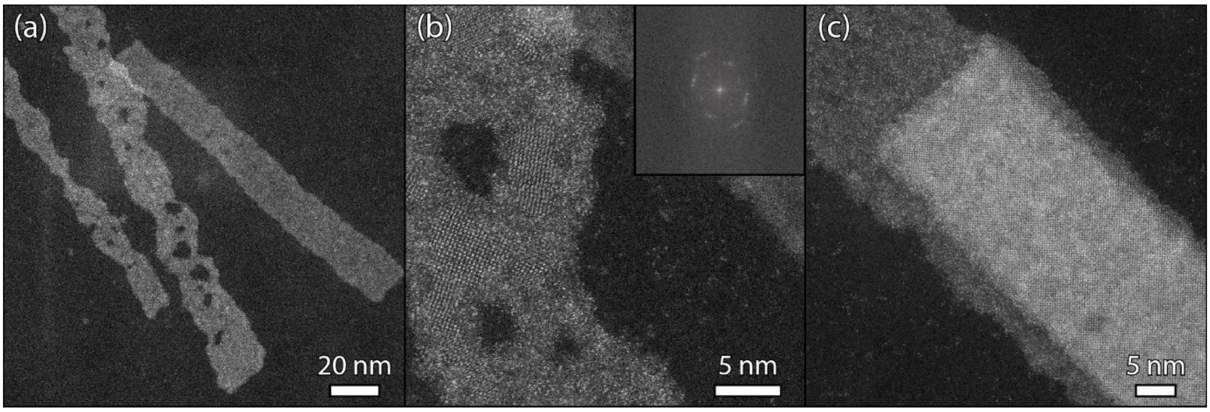

Figure S12. PbS nanosheets prepared at 145 °C for 2.5 minutes of reaction time. (a) Several NSs are partially "broken", having holes in the structure and misaligned edges. (b) The crystalline areas observed in "broken" NSs are still aligned with the [010] direction in the long lateral dimension of the NSs. (c) Two or more amorphous-like NSs located on top of each other show more crystallinity.

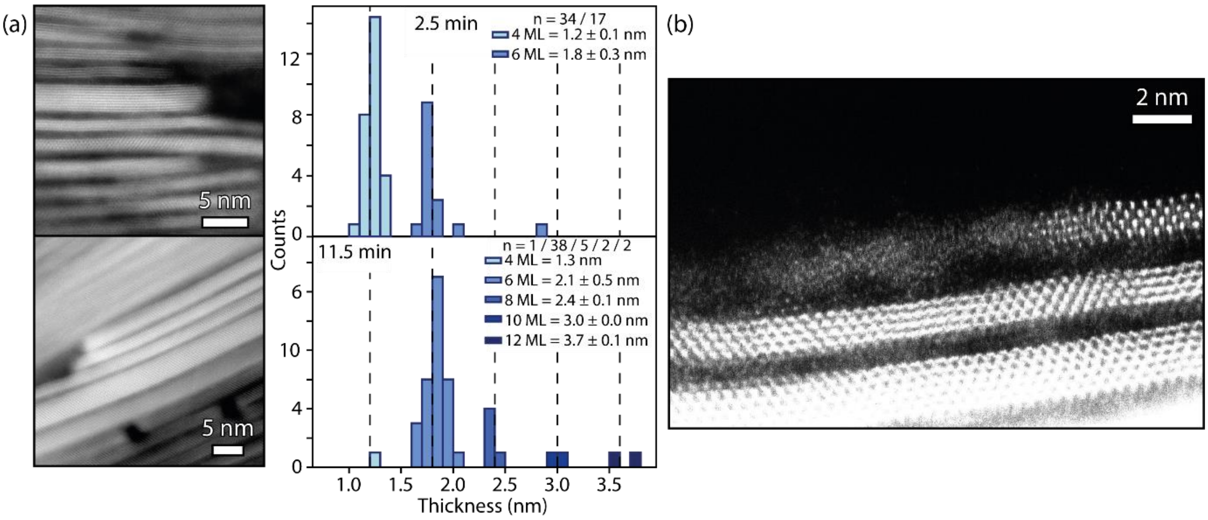

Figure S13. Determination of the thickness of PbS NSs prepared at 145 °C after 2.5 and 11.5 minutes (final product) after the sheets were stacked by the addition of an anti-solvent. (a) HAADF-STEM images and histograms of the measured NSs. In the earliest aliquot, 4 and 6 MLs NSs are observed in a 2:1 ratio. In the final product most of the sheets are 6 MLs thick, but a small number of thicker NSs (8, 10 and even 12 MLs) are observed in small numbers. (b) Shows the influence the addition of anti-solvent can have on a less stable sheet, potentially a pseudo-crystalline NS such as in Figure 6b. This provides an indication that all the sheets measured in panel (a) are the ones stable enough to stack after anti-solvent addition. Thus, it is possible that the results in panel (a) show the more stable sheets in the dispersion i.e. sheets that are more crystalline and thus mechanically more stable.

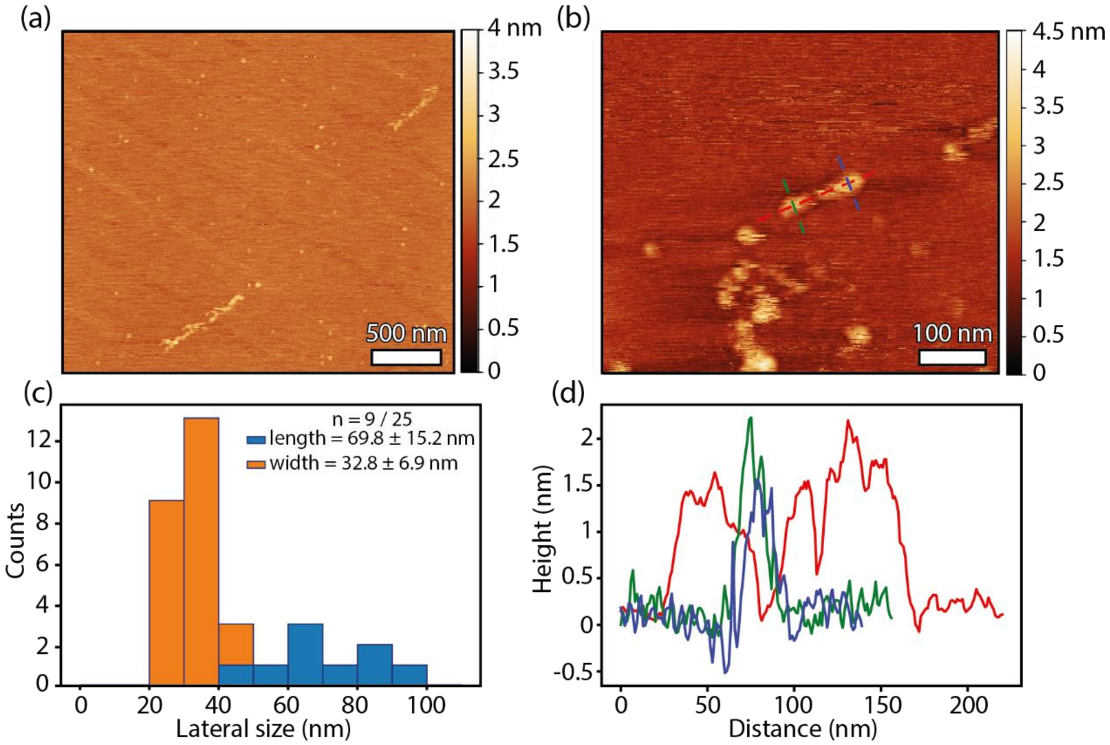

Figure S14. In an attempt to determine the thickness of the NSs in the first aliquot of the 145 °C reaction without the addition of an anti-solvent we used atomic force microscopy (AFM). The dispersion was dropcasted on a cleaved mica and AFM was used to image the sheets. Due to clustering and some contamination the sheets are imaged as elongated with round edges (a and b). (c) Histogram of the length and width AFM measurements is shown. The width of the sheets corresponds well to TEM measurements, but due to clustering of the NSs the length is challenging to measure and the resulting value is quite low. (d) Some representative height measurements are shown, both 1.8 nm (green, 6 MLs) and 1.2 nm (red, 4 MLs) are observed, although here we observe even some thinner 0.8 nm (blue). This third population is however slightly thicker than expected, if these are indeed sheets of 2 MLs we would expect 0.6 nm. See Figure S15a for the histogram of the measurements.

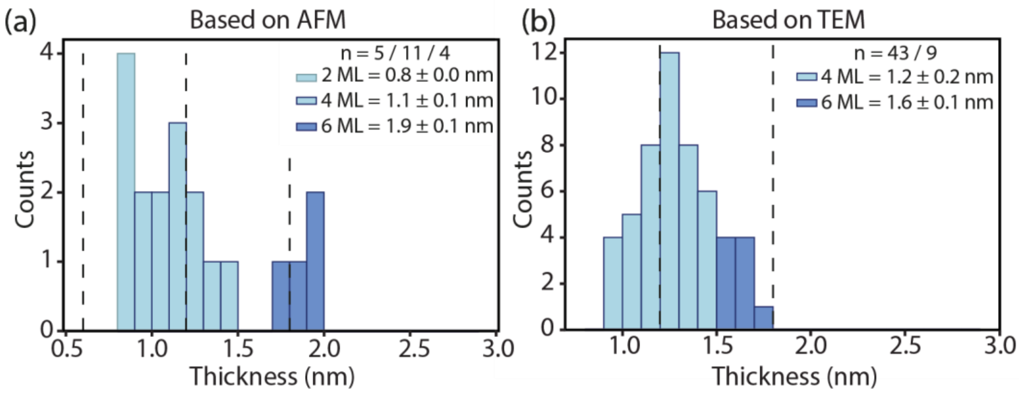

Figure S15. Histograms of the thickness of the first sample grown at 145°C. The AFM measurements show on average thinner sheets than TEM measurements of both the NSs stacked by antisolvent addition (Figure S13) and stacks naturally occurring in the sample (b). However, the number of sheets measured by AFM is too low to draw any definitive conclusions.

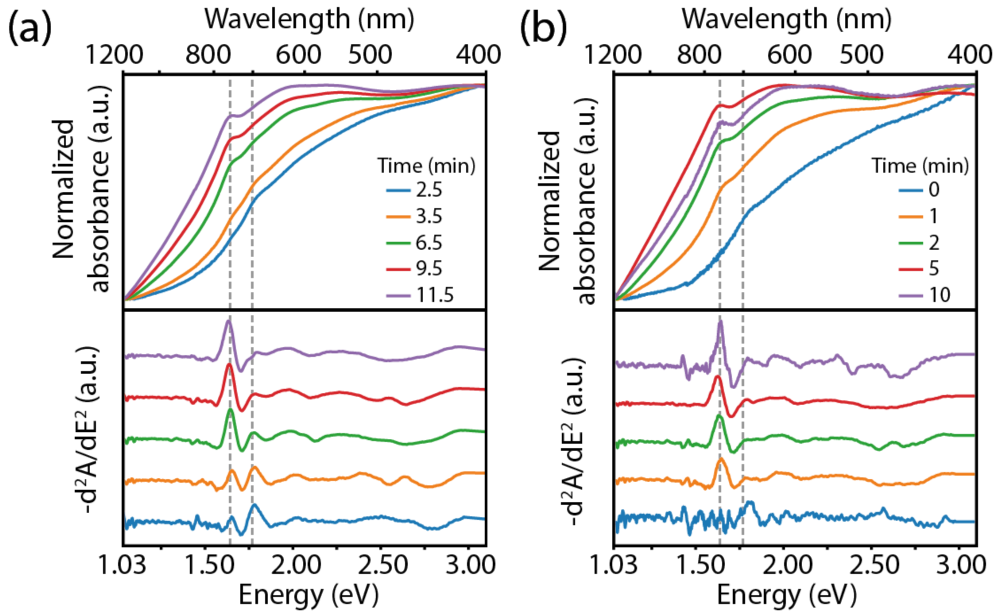

Figure S16. Absorption spectra of aliquots from PbS NSs dispersions prepared at 145°C (a) and 160°C (b, top); together with corresponding second-derivative analysis (below). The dashed vertical lines are located at respectively 1.64 and 1.78 eV. In both experiments a shift from 1.78 eV to 1.64 eV is observed over time, indicating an increase in thickness from 4 MLs to 6 MLs.

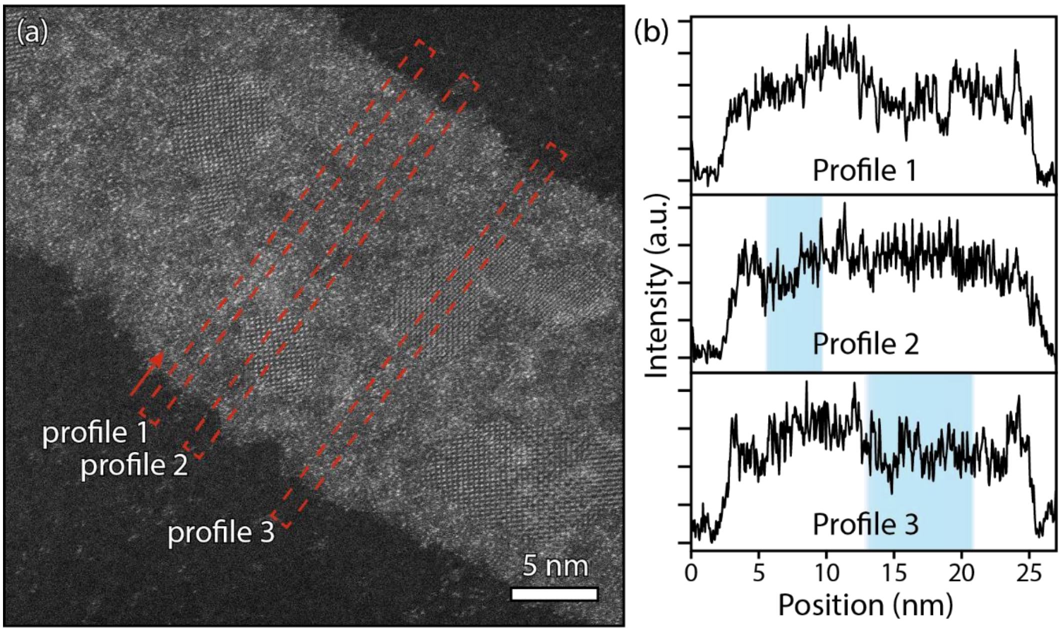

Figure S17. To investigate possible thickness differences between the crystalline (rock salt and orthorhombic) and pseudo-crystalline areas of a PbS NSs prepared at 145 °C for 2.5 minutes of reaction, intensity profiles were taken across the NS. (a) High-resolution HAADF-STEM image showing crystalline domains in the pseudo-crystalline structure. (b) Intensity profiles along the corresponding dashed red regions in panel (a). The blue shaded areas correspond to crystalline domains along the profile. No clear relation is observed between the intensity of a crystalline area versus that of a pseudo-crystalline area, indicating a homogeneous thickness in the NSs.

Section S3. Formation mechanism of the PbS NSs

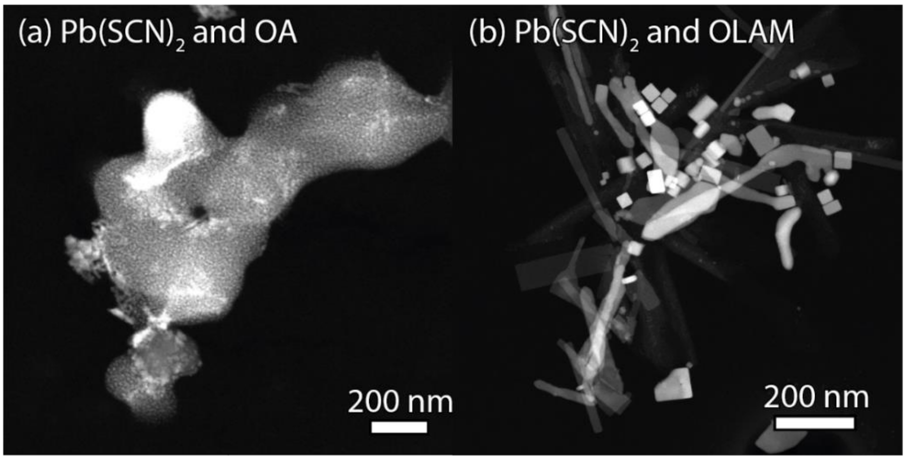

Figure S18. To investigate the role of oleic acid (OA) and oleylamine (OLAM) in the $Pb(SCN)_2$ decomposition reaction, OLAM and OA were replaced with an equal amount of respectively OA (a) and OLAM (b). (a) Replacement of OLAM with OA yields amorphous structures. (b) Replacement of OA with OLAM results in PbS cubes and two-dimensional structures, showing that OLAM is essential for the growth of PbS NSs. Similar influence of OLAM has been shown in dithiocarbamate single source precursor reactions.[9]

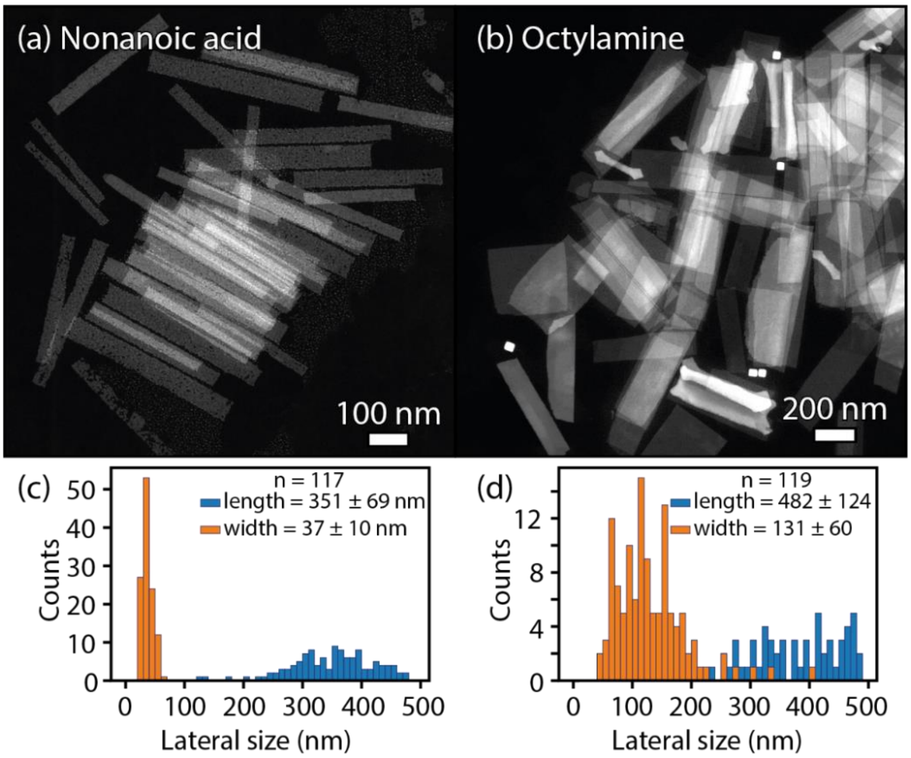

Figure S19. (a) Replacing OA with an equivalent amount of nonanoic acid results in PbS NSs with lateral dimensions of 351 ± 69 by 37 ± 10 nm. (b) Replacing OLAM for an equivalent of octylamine results in PbS NSs with lateral dimensions of 482 ± 124 by 130 ± 60 nm.

Section S4. Thickness and optical properties of annealed NSs

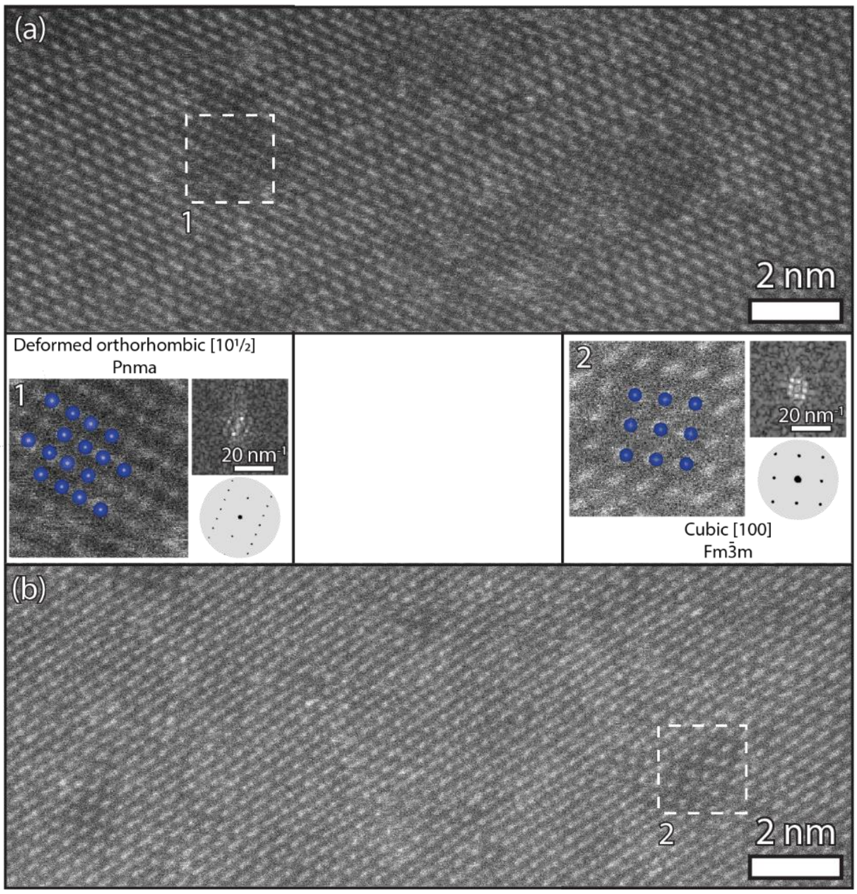

Figure S20. High-resolution HAADF-STEM images and Fourier transform of specific domains (see also Figure 4). The corresponding Fourier transforms from the various defects in the 5 minute (a) and 10 minute (b) grown sample were studied. In addition to the deformed orthorhombic crystal structure and previously discussed defects in the 5 minute sample, we observed deformed orthorhombic domains with different orientation (area 1). In the 10 minute sample, most of the defects have the cubic crystal structure.

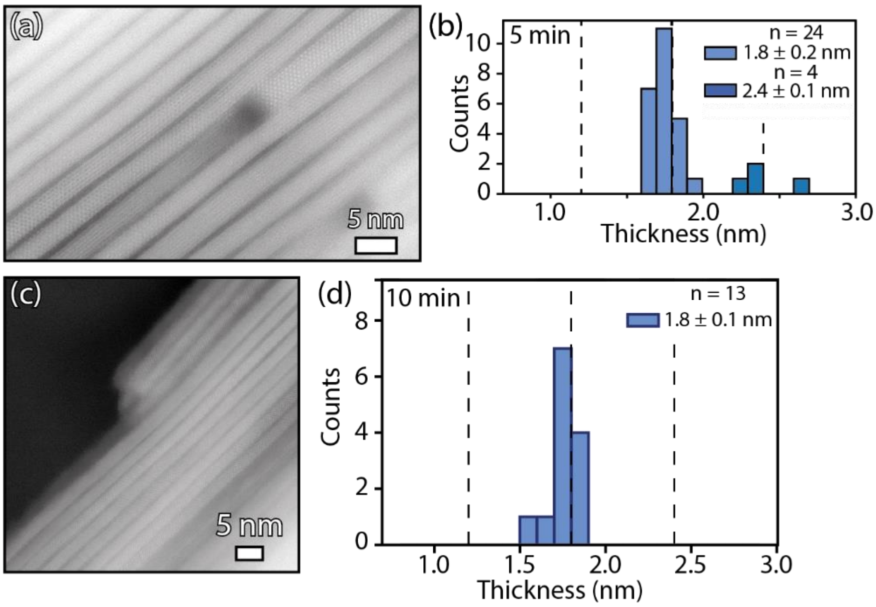

Figure S21. Determination of the thickness of PbS NSs with an edge-up orientation, prepared at 165 °C for 5 and 10 minutes. (a) High-resolution HAADF-STEM image showing NSs with thicknesses of 6 MLs and 8 MLs of PbS. (b) Corresponding histogram, indicating the absence of 1.2 nm sheets, but the presence of NSs with a thickness of 1.8 and 2.4 nm. (c) High-resolution HAADF-STEM image showing NSs with thicknesses of 6 MLs of PbS. (d) Corresponding histogram, indicating the absence of 1.2 nm sheets, but the presence of NSs with a thickness of 1.8 nm. It should be noted that the number of measured NSs for this characterization is quite low.

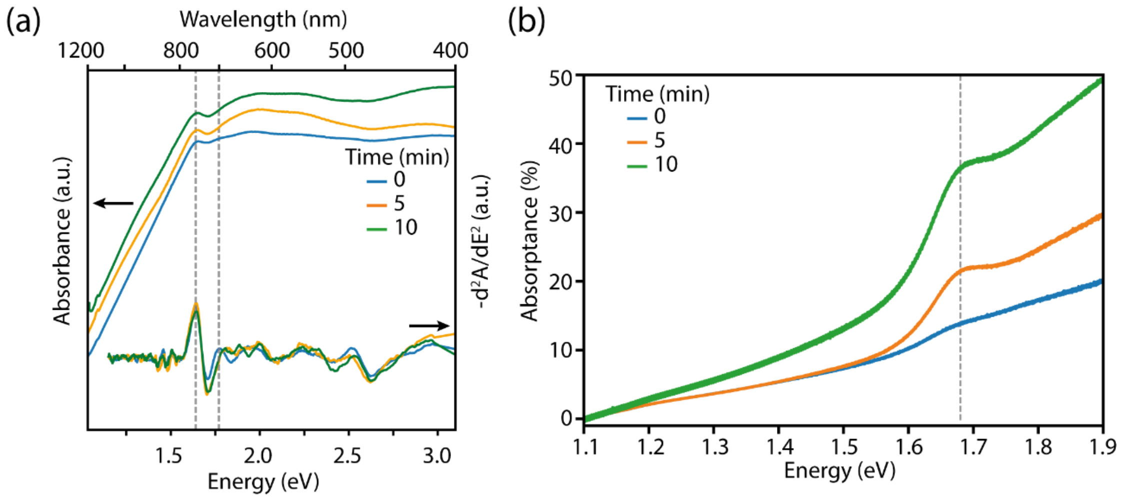

Figure S22. (a) The absorption spectra of PbS NSs prepared at 165 °C for 0, 5 and 10 minutes show an increase in background scattering while the absorption transition becomes more pronounced (1.64 and 1.77 eV dashed vertical lines). Analysis with the second-derivative shows a transition at 1.64 eV, indicating that the NSs predominantly attained a thickness of 6 MLs. Hence, prolonged reaction at 165 °C induces a thickness increase from 4 to 6 MLs, resulting in a more uniform population of NSs with a more homogeneous deformed orthorhombic crystal structure. (b) As per recent publications in PbS NSs films an absorptance of 4.6% (2πα) per independent layer of PbS NSs is expected.[10,11] The films were prepared by dropcasting 50 μL of the PbS NSs onto a quartz slide. At longer reaction times the absorptance increases from ~10 to 40%, which is attributed to an increase in NS concentration. The dashed vertical line is located at 1.68 eV.

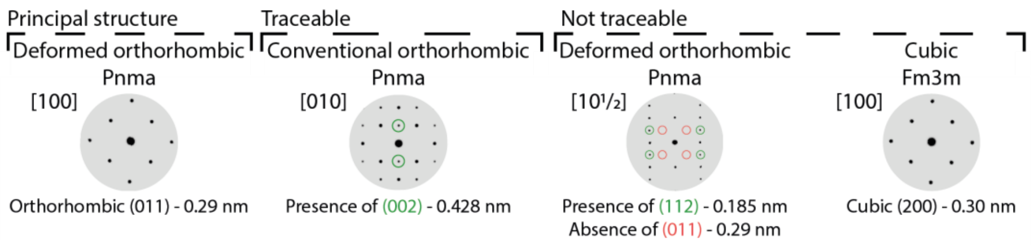

Figure S23. Schematic representation of the diffraction patterns of the principal deformed orthorhombic crystal structure and all the defects characterized in the NSs. Considering that the dumbbell atom columns of the deformed orthorhombic structure give the NSs higher intensity in the HAADF-STEM images with respect to the defect areas, we can apply intensity threshold image analysis (Figure 8). The diversity of the structures identified as defects, as well as their similarity in the diffraction diagrams (see schematic on the right) makes the analysis of these domains non-trivial. However, 4D STEM allows us to make a general interpretation of the diffraction patterns to gain knowledge in the homogeneity of the crystal orientation in the sheets.

References

(1) Macias-Pinilla, D. F.; Echeverría-Arrondo, C.; Gualdrón Reyes, A. F.; Agouram, S.; Muñoz-Sanjosé, V.; Planelles, J.; Mora-Seró, I.; Climente, J. I. Morphology and Band Structure of Orthorhombic PbS Nanoplatelets: An Indirect Band Gap Material. *Chem. Mater.* **2021**, *33* (1), 420-429. DOI: 10.1021/acs.chemmater.0c04281.

(2) Akkerman, Q. A.; Martín-García, B.; Buha, J.; Almeida, G.; Toso, S.; Marras, S.; Bonaccorso, F.; Petralanda, U.; Infante, I.; Manna, L. Ultrathin Orthorhombic PbS Nanosheets. *Chem. Mater.* **2019**, *31* (19), 8145-8153. DOI: 10.1021/acs.chemmater.9b02914.

(3) Toso, S.; Baranov, D.; Altamura, D.; Scattarella, F.; Dahl, J.; Wang, X.; Marras, S.; Alivisatos, A. P.; Singer, A.; Giannini, C.; et al. Multilayer Diffraction Reveals That Colloidal Superlattices Approach the Structural Perfection of Single Crystals. *ACS Nano* **2021**, 6243–6256. DOI: 10.1021/acsnano.0c08929.

(4) Moreels, I.; Fritzinger, B.; Martins, J. C.; Hens, Z. Surface Chemistry of Colloidal PbSe Nanocrystals. *J. Am. Chem. Soc.* **2008**, *130* (45), 15081-15086. DOI: 10.1021/ja803994m.

(5) De Roo, J.; Yazdani, N.; Drijvers, E.; Lauria, A.; Maes, J.; Owen, J. S.; Van Driessche, I.; Niederberger, M.; Wood, V.; Martins, J. C.; et al. Probing Solvent–Ligand Interactions in Colloidal Nanocrystals by the NMR Line Broadening. *Chem. Mater.* **2018**, *30* (15), 5485-5492. DOI: 10.1021/acs.chemmater.8b02523.

(6) Singh, S.; Leemans, J.; Zaccaria, F.; Infante, I.; Hens, Z. Ligand Adsorption Energy and the Postpurification Surface Chemistry of Colloidal Metal Chalcogenide Nanocrystals. *Chem. Mater.* **2021**, *33* (8), 2796-2803. DOI: 10.1021/acs.chemmater.0c04761.

(7) Anderson, N. C.; Hendricks, M. P.; Choi, J. J.; Owen, J. S. Ligand Exchange and the Stoichiometry of Metal Chalcogenide Nanocrystals: Spectroscopic Observation of Facile Metal-Carboxylate Displacement and Binding. *J. Am. Chem. Soc.* **2013**, *135* (49), 18536-18548. DOI: 10.1021/ja4086758.

(8) Pennycook, T. J.; McBride, J. R.; Rosenthal, S. J.; Pennycook, S. J.; Pantelides, S. T. Dynamic Fluctuations in Ultrasmall Nanocrystals Induce White Light Emission. *Nano Lett.* **2012**, *12* (6), 3038-3042. DOI: 10.1021/nl3008727.

(9) Zhang, Y.; Lu, J.; Shen, S.; Xu, H.; Wang, Q. Ultralarge Single Crystal SnS Rectangular Nanosheets. *Chem. Commun.* **2011**, *47* (18), 5226-5228. DOI: 10.1039/c0cc05528j.

(10) Prins, P. T.; Alimoradi Jazi, M.; Killilea, N. A.; Evers, W. H.; Geiregat, P.; Heiss, W.; Houtepen, A. J.; Delerue, C.; Hens, Z.; Vanmaekelbergh, D. The Fine-Structure Constant as a

Ruler for the Band-Edge Light Absorption Strength of Bulk and Quantum-Confined Semiconductors. *Nano Lett.* **2021**, *21* (22), 9426-9432. DOI: 10.1021/acs.nanolett.1c02682.
(11) Lannoo, M.; Prins, P. T.; Hens, Z.; Vanmaekelbergh, D.; Delerue, C. Universality of Optical Absorptance Quantization in Two-Dimensional Group-IV, III-V, II-VI, and IV-VI Semiconductors. *Phys. Rev. B* **2022**, *105* (3), 035421. DOI: 10.1103/PhysRevB.105.035421.
